# Genome-Wide Identification and Analysis of Apple NITRATE TRANSPORTER 1/PEPTIDE TRANSPORTER Family (NPF) Genes Reveals MdNPF6.5 Confers High Capacity for Nitrogen Uptake under Low-Nitrogen Conditions

**DOI:** 10.3390/ijms19092761

**Published:** 2018-09-14

**Authors:** Qian Wang, Changhai Liu, Qinglong Dong, Dong Huang, Cuiying Li, Pengmin Li, Fengwang Ma

**Affiliations:** State Key Laboratory of Crop Stress Biology for Arid Areas/Shaanxi Key Laboratory of Apple, College of Horticulture, Northwest A & F University, Yangling 712100, China; wangqian123@nwafu.edu.cn (Q.W.); chliu@nwafu.edu.cn (C.L.); dong19850412@163.com (Q.D.); Mrhaodee@126.com (D.H.); lcy1262@nwsuaf.edu.cn (C.L.)

**Keywords:** apple, NPF gene family, genome-wide, nitrate concentration, expression analysis

## Abstract

The NITRATE TRANSPORTER 1/PEPTIDE TRANSPORTER family (NPF) proteins play important roles in moving substrates such as nitrate, peptides, amino acids, dicarboxylates, malate, glucosinolates, indole acetic acid (IAA), abscisic acid (ABA), and jasmonic acid. Although a unified nomenclature of NPF members in plants has been reported, this gene family has not been studied as thoroughly in apple (*Malus* × *domestica* Borkh.) as it has in other species. Our objective was to provide general information about apple *MdNPF*s and analyze the transcriptional responses of some members to different levels of nitrate supplies. We identified 73 of these genes from the apple genome and used phylogenetic analysis to organize them into eight major groups. These apple NPFs are structurally conserved, based on alignment of amino acid sequences and analyses of phylogenetics and conserved domains. Examination of their genomic structures indicated that these genes are highly conserved among other species. We monitored 14 cloned *MdNPF*s that showed varied expression patterns under different nitrate concentrations and in different tissues. Among them, *NPF6.5* was significantly induced by both low and high levels of nitrate. When compared with the wild type, *35S:MdNPF6.5* transgenic apple calli were more tolerant to low-N stress, which demonstrated that this gene confers greater capacity for nitrogen uptake under those conditions. We also analyzed the expression patterns of those 73 genes in various tissues. Our findings benefit future research on this family of genes.

## 1. Introduction

Uptake, transport, and recycling of nutrients are critical processes during the plant life cycle. Nitrogen is a major component of proteins, nucleic acids, cell walls, phospholipids, chlorophyll, hormones, vitamins, enzymes/coenzymes, and alkaloids [1]. A series of pathways, including transporters and ion channels, direct nitrate uptake from the soil, its long-distance transport, source-to-sink allocations, homeostasis, and signal transduction [1,2] have been reported. These nitrate and peptide transporters have important roles in nutrient cycling [3,4,5]. Nitrate is a valuable source of nitrogen (N) for higher plants, especially in arid and semi-arid regions [6,7]. Through various mechanisms, a large part of the nitrate is absorbed from the soil by nitrate transporters (NRTs), e.g., NRT1/PTR, NRT2, and NRT3. When adapting to changing concentrations of soil nitrate, plant roots utilize different systems of absorption, including a low-affinity transport system (LATS, >1 mM) and a high-affinity transport system (HATS, 1 μM–1 mM). Two types of transportation are used—constitutive (cLATS/cHATS) and inducible (iLATS/iHATS)—that are determined by whether gene expression can be induced by a particular soil nitrate concentration [1]. The first discovered NRT member was AtNRT1.1 or CHL1 in *Arabidopsis thaliana* (hereafter, *Arabidopsis*). This dual-affinity nitrate transporter has a very wide absorption range for both high and low concentrations of nitrate [8]. It also plays a valuable role in nitrate transport from roots to stems as well as in nitrogen-regulated auxin transport and root morphology [9]. In *Arabidopsis*, *NRT1.5* is a bi-directional transporter that is critical for the influx and efflux of root-to-shoot translocation of nitrate [10]. *AtNRT1.6* is mainly responsible for moving nitrate to seeds to support their development [11], while *AtNRT1.8* and *AtNRT1.9* have roles in long-distance transport and in the xylem-to-phloem process of nitrate-loading [1].

NRT1 belongs to the peptide transporter (PTR) family, members of which are composed of dipeptide and tripeptide transporters that act as proton-dependent oligo peptide transporters (POTs) in plants [12,13,14]. The PTR family can be divided into several groups according to differences in substrates, with some members, such as those within the NRT subfamily, being involved in nitrate transport. All PTRs share a strong conserved sequence and 12 putative transmembrane (TM) regions, including a large hydrophilic loop between TM domains 6 and 7. Members of this PTR family tend to have 450–600 amino acids (aa). Substrate specificity means that members are classified into one of three types: di-/tripeptide transporter, nitrate transporter, or other substrate transporter [15]. The first di-/tripeptide transporter member, AtPTR2, was identified in *Arabidopsis*, and shows relatively higher expression levels in certain organs and at different developmental stages, e.g., three-day-old germinants, seedling roots, and young leaves [13]. In rice (*Oryza sativa*; Os), most OsPTR members have three highly conserved motifs [14]. Although located at different chromosomal positions, the AtPTR family members in *Arabidopsis* also have three conserved motifs [14]. The plant PTR family is thought to have key roles in nitrogen metabolism, tolerance to abiotic stresses, and the seed development. For example, *AtPTR3* confers tolerance to NaCl stress and infections by bacterial pathogens [16,17]. Expression of *AtPTR5* promotes the accumulation of peptides in pollen, ovules, and developing seeds [18].

Because NRT1 and PTR are related, the unified family—NITRATE TRANSPORTER 1/PEPTIDE TRANSPORTER (NRT1/PTR)—is named NPF, a label now used in the phylogenetic trees of 33 fully sequenced plant genomes [19]. Plant NPF proteins can transport several types of substrates, such as nitrate [15], peptides [14], dicarboxylates [20], glucosinolates [21], indole acetic acid (IAA) [9], abscisic acid (ABA) [22], and gibberellin (GA) [23]. All NPFs in higher plants share high similarity among sequences and contain 12 putative TM regions connected by short peptide loops. In between each group of six TM regions is a large hydrophilic loop. Phylogenetic analysis of NPFs in 33 fully sequenced genomes has shown that this family can be divided into eight well-defined subfamilies.

In Arabidopsis, *AtNPF6.2* and *AtNPF6.3* play major roles in nitrate uptake at high concentrations and *AtNPF6.2* is also a low-affinity nitrate transporter [2]. *AtNPF1.1* and *AtNPF1.2* are more highly expressed in expanded leaves, where nitrate is transferred between xylem and phloem for optimal distribution [24]. Some sources of stress, including phytohormones ethylene and jasmonate, regulate the expression of *AtNPF7.2* and *AtNPF7.3*, causing nitrate to accumulate in the roots [25]. Both *AtNPF2.12* and *AtNPF5.5* are critical in the transport of sufficient nitrate to developing seeds [26]. *VvNPF3.2* is a pathogen-inducible transporter in *Vitis vinifera*. Some NPF genes in potato (*Solanum tuberosum*) are up-regulated when plants are infected by potato virus Y (PVY), which suggests that nutrient transport can enhance plant tolerance to PVY [27,28].

Several family members with highly conserved NPF domains have been identified in many plant species, including *Arabidopsis* [29,30], rice [31,32], *Triticum aestivum* [33], poplar (*Populus trichocarpa*) [34], *Lotus japonicas* [35], tomato (*Solanum lycopersicum*) [36], and *Catalpa bungei* [37]. However, only a few systematic analyses have been conducted for apple NPF genes. Here, we examined their protein and gene structures, conserved domains, phylogenetic relationships, chromosomal locations, and TM regions. We also assessed their expression in various tissues (roots, stems, leaves, flowers, and fruit) and 14 of them in response to different nitrate concentrations. As the first systematic study of this family in apple, our results will provide a valuable basis for selecting candidate genes to improve the efficiency of nitrogen utilization and further investigating the function of *MdNPF*s in that fruit crop.

## 2. Results

### 2.1. Identification and Annotation of NPF Genes in Apple

To identify the NPF genes in apple, we conducted a Blast P against its genome database. According to the 139 sequences previously identified by Léran et al. [19], 89 *NPF*s were retained after removing the same sequences or new sequences in the Md3.0 version for genome annotation. From those 89, 16 were then deleted because their sequences were too short or too long (Supplementary Materials, Table S1. The nomenclature of the apple NPF genes followed previously published rules, i.e., the name should be NPFX.Y, where X represents the subfamily and Y stands for the specific member within the subfamily [19]. From this, we summarized details including the chromosome location and ORF of each gene, as well as the protein length, molecular weight, and theoretical isoelectric point (pI) for each protein that an *MdNPF* encoded (Table 1). Each apple *NPF* usually encoded 400–600 aa, with molecular weights ranging from 29.89 to 76.60 kDa. The theoretical pI was distributed between 5.26 and 9.62, mainly between 6.00 and 7.00.

### 2.2. Phylogenetic Tree of NPF in Apple

**Figure 1:**
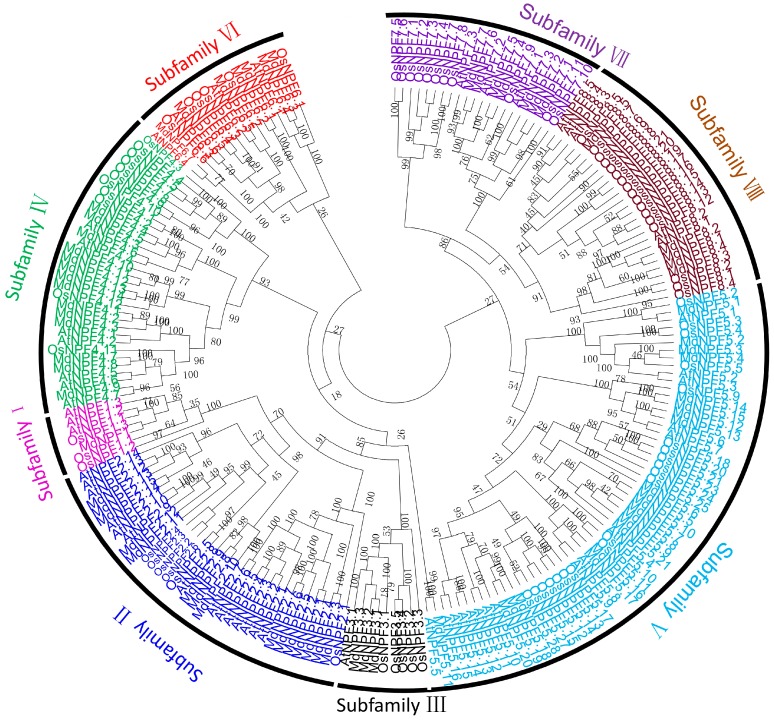
Phylogenetic tree and subfamily information for MdNPFs, AtNPFs, and OsNPFs. Neighbor-Joining method was used in tree construction with MEGA 5 software for 205 full-length amino acid sequences from apple, *Arabidopsis*, and rice. Eight subfamilies are indicated with Roman numerals. The numbers at nodes of the phylogenetic tree indicate the bootstrap values expressing branching probability per 1000 replicates; the bootstrap values of the confidence levels are shown as percentages.

We examined the phylogenetic relationship and function divergence of MdNPF genes by constructing a phylogenetic tree for protein sequences for 73 of them. This tree showed that the *MdNPF*s could be divided into eight major clades (I–VIII) according to the unified nomenclature. Each clade was considered to be one sub-family. To identify the order of every gene within a subfamily, we gave a second number to each gene. Evolutionary analysis suggested that the eight subfamilies in apple were similar to those found in *Arabidopsis* and rice (Figure 1).

Subfamilies I and II were more closely related to each other, as were subfamilies VI and VIII. Subfamilies I–VIII contained 1, 15, 3, 14, 21, 7, 7, and 5 members, respectively. The 15 NPF members in subfamily II were further divided into two groups (Figure 1).

### 2.3. Chromosomal Localization Analysis of NPFs in Apple

**Figure 2:**
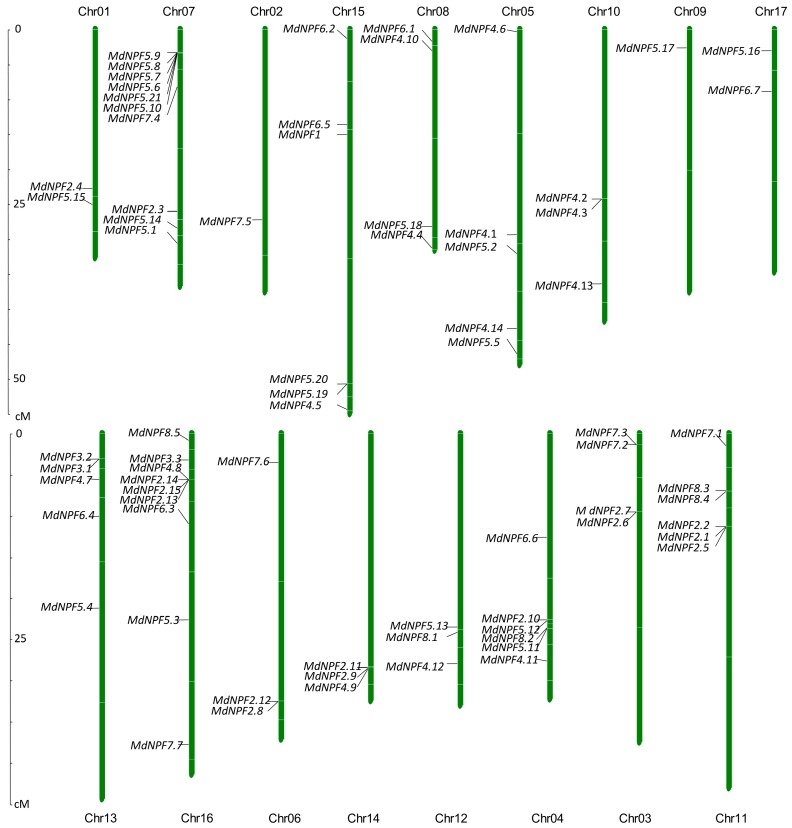
Chromosome positions for MdNPF genes, marked with solid black lines. Scale on left is in Mb. Chromosome numbers are indicated on top of bar.

We confirmed the chromosomal location of each *NPF* according to mapping coordinates for the apple genomic sequence. The 73 *MdNPF*s were distributed unevenly on 17 apple chromosomes, with Chromosome (Chr) 07 containing 10 genes (*MdNPF5.9*, *MdNPF5.8*, *MdNPF5.7*, *MdNPF5.6*, *MdNPF5.21*, *MdNPF5.10*, *MdNPF7.4*, *MdNPF2.3*, *MdNPF5.14*, and *MdNPF5.1*), Chr16 having nine (*MdNPF8.5*, *MdNPF3.3*, *MdNPF4.8*, *MdNPF2.14*, *MdNPF2.15*, *MdNPF2.13*, *MdNPF6.3*, *MdNPF5.3*, and *MdNPF7.7*), and Chr02 and Chr09 each having one, i.e., *MdNPF7.5* and *MdNPF2.17*, respectively (Figure 2).

Certain genes were closely aligned on the chromosomes, such as *MdNPF5.9*, *MdNPF5.8*, *MdNPF5.7*, and *MdNPF5.6* on Chr07; *MdNPF4.2* and *MdNPF4.3* on Chr10; and *MdNPF8.3* and *MdNPF8.4* on Chr11. The distribution pattern of various genes revealed that a particular region of a chromosome or certain chromosomes had a relatively higher density. Their sequence lengths and genetic structure were very similar, which may have indicated serial replication within the apple NPF family.

### 2.4. Analyses of Conserved Domains and TM Regions

**Figure 3:**
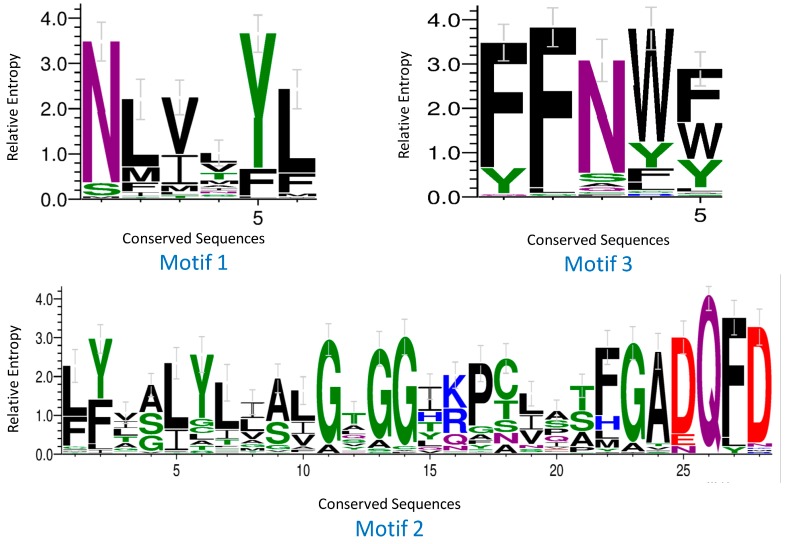
Sequence analysis of conserved domains from apple NPF proteins. *X*-axis, sequence of conserved motif; *Y*-axis, relative entropy that reflects rate of conservation for each amino acid.

Protein sequence analysis demonstrated that each apple NPF contained a complete, conserved NPF domain. The MdNPFs generally possessed 12 TM regions (400–600 aa) that were connected by short peptide loops. A large hydrophilic loop (approximately 100 aa) occurred between the sixth and seventh TM region in each gene (Supplementary Materials, Figure S1. After aligning the protein sequences, we detected three highly conserved motifs in most of the MdNPFs. Motif 1 (NLVxYL) was found between the first and second TM region; Motif 2 (LYxxLYLxALGxGGxK(R)PCxxXFGADQFD) in the fourth TM region; and Motif 3 (FFNWF) at the beginning of the fifth TM region (Figure 3).

### 2.5. Comparison of Exon‒Intron Structures for NPF Genes in Apple and Other Species

**Figure 4:**
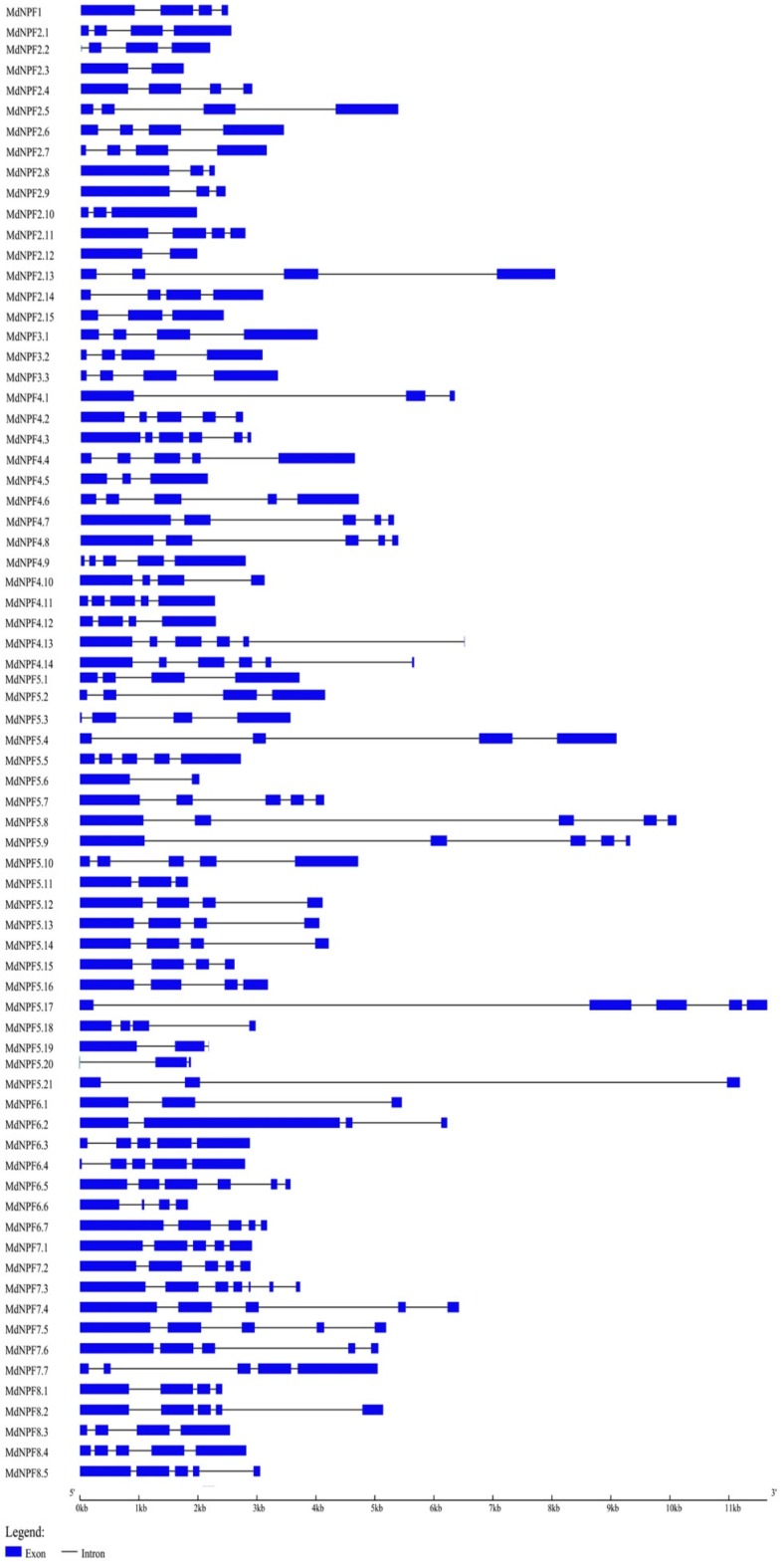
Structure analysis of apple *MdNPF* family. Rectangle filled with blue, exon; solid black line, intron. Scale at bottom is in kb.

**Figure 5:**
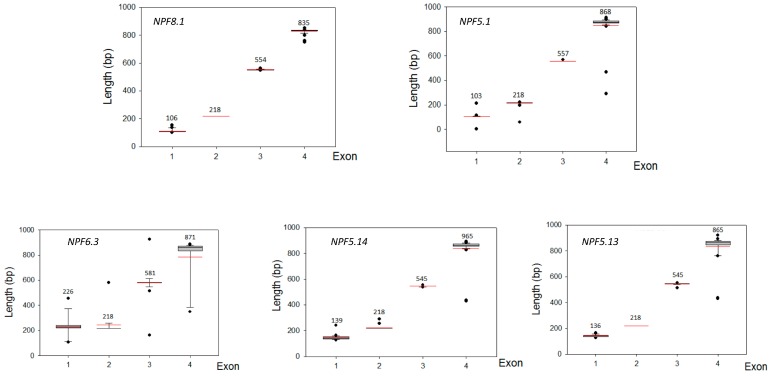
Exon-length distribution for *NPF5.1*, *NPF6.3*, *NPF5.13*, *NPF5.14*, and *NPF8.1* in different plant species. Analysis was based on Boxplot depictions in SigmaPlot 12.0 program. Each box represents exon size range in which 50% of values for particular exon are grouped. Mean value is indicated by long red line.

We analyzed the exon‒intron organization of coding sequences for *MdNPF*s and those genes in some other species. The structures were mapped according to the exon location and gene length of the coding regions (Figure 4). Within the eight clades of *MdNPF*s, the number of exons was not evenly distributed, but ranged from two to seven. In total, 29 genes (40% of all *MdNPF*s) had four exons each. In particular, all members of subfamilies I and III contained four exons. Twenty-five genes, mainly in subfamilies IV, V, VII, and VIII, had five exons. The exception was *MdNPF7.3*, which was the only gene containing seven exons. Genes containing six exons appeared only in subfamilies IV and VI, and included *MdNPF4.3*, *MdNPF4.13*, *MdNPF4.14*, and *MdNPF6.5*. Three other members in subfamilies II and V—*MdNPF2.3*, *MdNPF2.12*, and *MdNPF5.6*—each had two exons. For the other species, the number of exons was highly consistent, with nearly all containing four exons each, including *NPF5.1*, *NPF5.13*, *NPF5.14*, *NPF6.3*, and *NPF8.1* (Figure 5 and Supplementary Materials, Table S2).

### 2.6. Analysis of Expression for 14 MdNPFs in Response to Different Nitrate Concentrations

**Figure 6:**
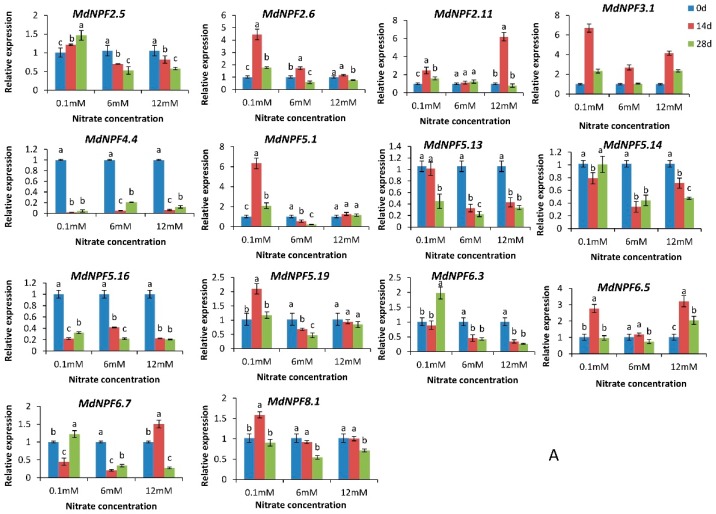

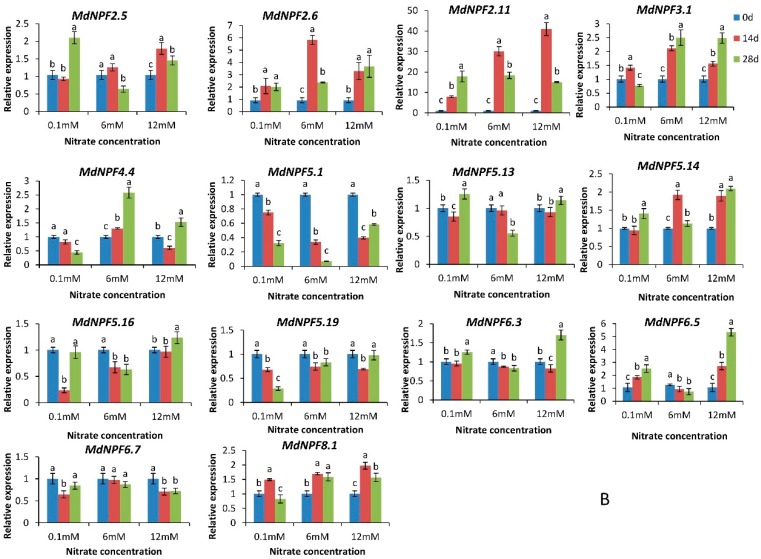
Relative expression levels for 14 cloned apple *NPF*s under different nitrate concentrations, calculated by 2^−ΔΔ*C*t^ method with respect to control samples (i.e., 6 mM NO_3_^−^): (**A**) the relative expression levels for 14 cloned apple *NPF*s of roots under different nitrate concentrations; and (**B**) the relative expression levels for 14 cloned apple *NPF*s of leaves under different nitrate concentrations. Different letters on the bars within a group indicate significant differences (*p* < 0.05), based on Tukey’s multiple range tests.

The *MdNPF*s were constitutively expressed in the roots, stems, leaves, flowers, and fruit, but transcription levels in specific tissues also varied according to developmental stage (Supplementary Materials, Figure S2). For functional analysis, we cloned 14 *MdNPF*s (Table 1) and monitored their expression profiles in response to different nitrate concentrations (Figure 6A). Whereas *MdNPF2.6*, *MdNPF3.1*, *MdNPF5.1*, and *MdNPF5.9* were induced by low-N treatment, *MdNPF2.11* and *MdNPF6.7* were up-regulated by high-N conditions when compared with the control plants. In addition, expression of *MdNPF3.1* and *MdNPF6.5* was up-regulated by both low-and high-nitrate concentrations in 14-day-old roots. When compared with the control, expression of *MdNPF3.1* and *MdNPF5.1* was up-regulated by almost seven-fold in response to low-N treatment.

In the leaves, *MdNPF2.5* and *MdNPF2.6* were up-regulated by more than two-fold under the low-nitrate concentration when compared with the control. Under high-N treatment, *MdNPF2.11*, *MdNPF5.14*, *MdNPF6.3*, and *MdNPF8.1* were up-regulated, with transcript levels of *MdNPF2.11* increasing by 40-fold. Expression of *MdNPF6.5* was up-regulated in both roots and leaves under either nitrate concentration (Figure 6B).

### 2.7. Effect of Low-Nitrogen Treatment on Growth by Apple Calli Tissue

**Figure 7:**
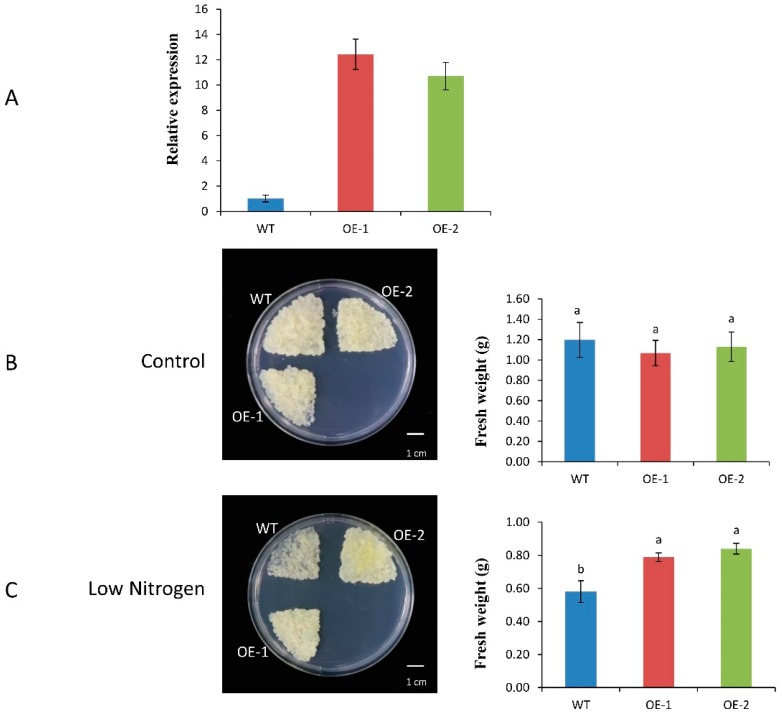
Influence of overexpression by *MdNPF6.5* on tolerance by apple calli to low-nitrogen supply. (**A**) Quantitative real time RT-PCR of samples from WT and *MdNPF6.5*-overexpressors. (**B**) Assay of low-nitrogen tolerance by WT and *MdNPF6.5*-overexpressors. Calli were transferred to MS medium or low-nitrogen medium, and photographed at 20 days after treatment began. (**C**) Comparison of fresh weights from WT and *MdNPF6.5*-overexpressors in response to low nitrogen. Values are means ± standard deviation. Different letters on the bars indicate significant differences (*p* < 0.05), based on Tukey’s multiple range tests.

Two lines of transgenic (*35S:MdNPF6.5*) apple calli showed relatively higher expression levels (inductions of 11- and 12-fold) when compared with the control (Figure 7A). Whereas growth rates on the MS media were similar among those overexpression calli and the WT (Figure 7B), their phenotypes differed between the control and transgenic lines when transferred to low-N MS media. Biomass production was also significantly greater from the transgenics than from the WT (Figure 7C).

## 3. Discussion

The NPF genes encode numerous proteins that comprise a large family of members broadly distributed in prokaryotes and eukaryotes [14,38]. As one of the most important fruit crops, apple is widely cultivated in China and around the world due to its high economic and nutritional value. Sequencing of the apple genome has facilitated the identification and analysis of putative apple gene families genome-wide. The encoded proteins include members of the DREB [39], MYB [40], MADS-box [41], PHT [42], RAD23 [43], UGTs [44], SnRK2 [45] and WRKY families [46,47]. Although *NPF*s have been identified in other species, this family is not as well-understood in apple.

The number of NPF family members varies greatly among species. For example, 51, 53, 68, 80, and 93 genes have been reported for *Capsella rubella* [19], *Arabidopsis* [15], poplar [48], *Medicago truncatula* [49] and rice [19], respectively. By comparison, the apple genome contains 139 NPF genes, making this family much more prominent there than in other species [19]. Using the latest database of apple (version Md3.0), we identified 89 *MdNPF*s and performed a comprehensive analysis with 73 of them. Examination of the entire genome sequence dataset, sequence alignments, and gene expression provided insight into the apple NPF family.

Our comparison of *NPF* members among various species revealed that some of those genes have disappeared while others have been duplicated. Such duplication plays a vital role in gene family evolution and diversity, which occurs via three main mechanisms: segmental duplication, tandem duplication, or retro-position. For example, rice contains *OsNRT1.1A* and *OsNRT1.1B*, both of which are simultaneously expressed, although the former is mainly expressed in the roots and has a higher transcription level than the latter [50]. Those two genes function similarly to *AtNRT1.1* from *Arabidopsis* [5]. In contrast, three *AtNRT1.1*-like genes found in grasses may have arisen as a consequence of either a single-based mutation or gene duplication following the dicot‒monocot split [50]. Poplar carries only one *AtNRT1.1*-like gene and no *AtNRT1.4*-like gene [5,50]. Although a degraded pseudogene version related to *NRT1.6* and *NRT1.7* exists in the genome of *Sorghum bicolor*, no ESTs have been found in any database for that species. The situation is similar for *Brachypodium* and *Zea mays* [50]. Therefore, we might hypothesize that these significant contrasts in *NRT* members between apple and other species is due to gene duplications and deletions in the apple genome, all of which have driven the evolution of *MdNPF*s to adapt to changes in soil nitrate concentrations.

The MdNPFs are highly and structurally conserved, based on our comprehensive analyses of amino acid sequence alignments, phylogenetics, and conserved domains (Figure 1 and Figure 3 and Supplementary Materials, Figure S1). Similar results have been reported for *Arabidopsis* [15], rice [14], legume plants such as *Medicago* [49,51], and poplar [48]. For example, apple *NPF2.11*, *NPF5.1*, *NPF5.13*, *NPF6.3*, and *NPF8.1* share the same exon‒intron structures and exon lengths with members found in other species. Some genes, e.g., *NPF5.1* and *NPF5.14*, have a different number of exons but which are all the same length, probably due to a split or merger during the evolutionary process (Supplementary Materials, Table S2). Consistent with previous findings by Léran et al. [19], our examination revealed that many MdNPF members contain 12 TM regions, with a large hydrophilic loop in the middle and six TMs at either side (Supplementary Materials, Figure S1). We also noted three conserved motifs during our analysis of conserved domains in the apple NPFs. Although rice PTRs also contain three motifs [14], two of their conserved domains differ slightly from those of apple. These findings suggest that the variability in amino acid residues outside the conserved domain might determine the different functions by MdNPF members.

In many species, the expression of some NPF genes can be induced by changes in soil nitrate concentrations [24], external K^+^ concentrations [52], or other factors [53]. For example, AtNPF6.3 (At NRT1.1) can have one of two *Km* values, depending upon the nitrate concentration. In *Arabidopsis*, when the level of nitrate is higher than 1 mM, *AtNPF6.3* can behave as a low-affinity transporter but can then switch to a high-affinity mode when that concentration goes below 1 mM, all due to the phosphorylation of intracellular threonine by kinase CIPK23 [54]. Both *ZmNPF7.10* and *OsNPF7.9* show increased relative expression in the presence of high K^+^ when compared with performance in response to a low-K^+^ concentration [52]. Some *Arabidopsis NPF*s, including *NPF1.1*, *NPF1.2*, *NPF2.3*, *NPF2.7*, *NPF2.9*, *NPF2.12*, *NPF2.13*, *NPF4.6*, *NPF5.5*, *NPF6.2*, *NPF7.2*, and *NPF7.3*, are strictly LATS genes [1]. Our study results indicated that the expression of *MdNPF*s in roots and leaves fell into one of three categories: Type I, responsive to low-N conditions; Type II, responsive to high-N conditions; or Type III, no concentration-related differences in response. In particular, the Type I genes were *MdNPF2.5*, *MdNPF2.6*, *MdNPF3.1*, *MdNPF5.1*, *MdNPF5.9*, and *MdNPF6.5*, while Type II included *MdNPF2.11*, *MdNPF5.14*, *MdNPF6.3*, *MdNPF6.5*, and *MdNPF8.1*. The remaining genes belonged to Type III. Consistent with our results, *NPF3.1* and *NPF5.14* in *Arabidopsis* are involved in the transport of NO_3_^−^ [1]. Expression of *MdNPF6.5* (*MdNRT1.1*) was elevated under both low- and high-N treatment, which suggested that this gene encodes a dual-affinity nitrate transporter such as AtNRT1.1 [54,55]. Therefore, all of these findings demonstrate that NPF genes have important physiological roles and are expressed at different levels depending upon the soil nitrate concentration.

As shown from our experiments, overexpression of *MdNPF6.5* can increase apple biomass production under low-N conditions. This is consistent with results from studies of *Arabidopsis* and rice [5,15]. Taken together, our research confirms that *MdNPF6.5* is a promising candidate gene for improving nitrogen uptake and the tolerance of apple plants to low nitrate supplies.

## 4. Materials and Methods

### 4.1. Identification of Apple NPF Genes

The *Arabidopsis* NPF family database was downloaded from the TAIR website (available online: http://www.arabidopsis.org/) [56]. Information about 53 *Arabidopsis* NPF proteins and the consensus protein sequences of the NPF domain was used for our BlastP search (available online: http://www.rosaceae.org/tools/ncbi_blast) against predicted apple proteins. We then searched all of those NPF sequences against the apple genome database (available online: https://www.rosaceae.org/gb/gbrowse/malus_x_domestica/) with HMMER v3.0 and BlastP [56]. The Pfam database (available online: http://pfam.sanger.ac.uk/search) and NCBI-Conserved Domain Search (NCBI-CDD; available online: http://www.ncbi.nlm.nih.gov/Structure/cdd/wrpsb.cgi) were used to confirm the reliability of those protein sequences [41].

### 4.2. Sequence Alignment and Phylogenetic Analysis

We used DNAMAN 6.0 (Lynnon Biosoft, San Ramon, CA, USA ) with default parameters to align the multiple sequences of 73 MdNPF protein sequences. A phylogenetic tree of the MdNPF gene family was constructed by MEGA 5.2 software (available online: http://www.megasoftware.net) and the Neighbor-Joining (NJ) method, bootstrapping with 1000 replicates. This analysis was based on the amino acid sequences of MdNPF proteins as well as NPF proteins from *Arabidopsis* and rice [57].

### 4.3. Analyses of Exon‒Intron Structure and Genome Distribution

Genomic sequences and distributions of chromosomes and NPF genes were downloaded from the apple genome database. Exon‒intron information for orthologs of *MdNPF5.1*, *MdNPF5.13*, *MdNPF5.14*, *MdNPF6.3*, and *MdNPF8.1* in various species were downloaded from PLAZA 3.0 (available online: http://bioinformatics.psb.ugent.be/plaza/) [58]. The exon‒intron structures of MdNPF genes were drawn by gene structure display server 2.0 (available online: http://gsds.cbi.pku.edu.cn/). A map of chromosomal positions was completed with MapInspect (available online: www.plantbreeding.wur.nl/UK/software_mapinspect.html) [41].

### 4.4. Sequence Logo and Prediction of TM Regions

Sequence logos for the conserved domains of MdNPFs were generated by the application WebLogo (available online: http://weblogo.threeplusone.com) [59]. We predicted the TM regions for MdNPFs by using TMHMM Server v.2.0 (available online: http://www.cbs.dtu.dk/services/TMHMM/).

### 4.5. Plant Materials and Nitrogen Treatments

To monitor gene expression, we conducted a hydroponics experiment during the growing season in 2017. Seedlings of *Malus hupehensis* were first cultured in the mixture of sand and soil, with a volume ratio 1:1, until they were 15 cm tall. They were then placed in a hydroponics environment to grow for three weeks in a 1/2 Hoagland nutrient solution consisting of 3.47 mM Ca(NO_3_)_2_·4H_2_O, 5.0 mM KNO_3_, 1.0 mM K_2_HPO_4_, 2.0 mM MgSO_4_·7H_2_O, 2.5 mM FeSO_4_·7H_2_O, 2.5 mM EDTA-Na, 0.046 mM H_3_BO_3_, 0.0067 mM MnCl_2_·4H_2_O, 0.00077 mM ZnSO_4_·7H_2_O, 0.00032 mM CuSO_4_·5H_2_O, and 0.00011 mM H_2_MoO_4_·H_2_O (pH 6.0). Afterwards, the seedlings were cultivated in a modified Hoagland nutrient solution containing either 0.01 (low-N) or 12 mM nitrate (high-N). As the control treatment, we used a 6 mM nitrate solution. Young roots and leaves were collected on Days 0, 14, and 28 of treatment to examine the effects of different nitrate concentrations on *MdNPF* expression. All samples were frozen immediately in liquid N_2_ and stored at −80 °C prior to RNA extraction.

*Agrobacterium*-mediated transformation of apple “Orin” calli tissue was performed by using the open reading frame (ORF) cDNA of *MdNPF6.5* and cloning into vector pBI121 to produce the overexpression construct. The callus tissue was genetically transformed as described by Hu et al. [60]. Following identification, the transgenic calli were cultured on 11/12 MS medium without nitrogen and 1/12 MS medium for low N treatment. Other growth conditions remained the same. Photographs were taken and fresh weights recorded after 20 days of N-deficient treatment.

### 4.6. Quantitative Real Time RT-PCR (qRT-PCR) and Gene-Cloning

Total RNA was extracted from frozen tissues with a Wolact^®^ Plant RNA Isolation kit (Vicband, Hong Kong, China). The first-strand cDNA was synthesized by adding 2 μg to the reaction mixture. For the qRT-PCR assays, reverse-transcription was performed with 1 μg of total RNA from each sample, followed by PCR-amplification of 1 μL of the product. Previously prepared cDNA was used for qRT-PCR assays conducted in a 20-µL reaction system that included 10 µL of SYBR^®^ Premix Ex Taq™ (TaKaRa, Kyoto, Japan) and used a QuantStudio^®^5 instrument (Life Technologies, Carlsbad, CA, USA) as described before [61].

The Primers used for quantitative real time RT-PCR amplifications are listed in Supplementary Materials, Table S3. The RT-PCR amplifications involved an initial 95 °C for 3 min; 40 cycles at 95 °C for 10 s, 58 °C for 30 s, and 72 °C for 15 s; 3 min at 72 °C; and 81 cycles of 7 s each that increased by an increment of 0.5 °C, from 55 °C to 95 °C. Three biological replicates were set up for each assay and the ΔCt values were calculated by using *MdMDH* as the endogenous control [62]. The values of relative quantification were calculated based on the 2^−ΔΔ*C*t^ method [63] and dissociation curve analysis was used to determine the specificity of the amplifications.

The PCR reaction conditions for gene-cloning were 32 cycles of 98 °C for 10 s, 60 °C for 10 s, and 72 °C for 2 min, followed by 2 min extension at 72 °C. Primers used for gene-cloning are shown in Supplementary Materials, Table S4.

### 4.7. Statistical Analysis

All data were analyzed with SPSS 16.0 software (IBM, Chicago, IL, USA). One-way ANOVA and Tukey’s tests were used to compare the results under different nitrate concentrations versus the control.

## 5. Conclusions

We identified 73 MdNPFs in the apple genome and determined their expression patterns that varied according to tissue type and concentration of nitrate in nutrient solution. These results provide new information that can be applied to further investigations into the functions of apple *NPF*s when plants are responding to changes in nitrate levels. In particular, *MdNPF6.5* shows potential for research efforts to improve tolerance to nitrogen deficiencies by apple and, possibly, other crops.

## Figures and Tables

**Table 1:** Basic information about apple NPFs.

Gene Name	Gene ID ^a^	Chromosome Location	ORF	Protein Length (aa)	MW (kDa)	Theoretical Isoeletrical Point (pI)	GenBank Accession Numbers of Cloned Gene
*MdNPF1*	MD15G1190800	Chr15:15008548-15011039	1779	593	65.21	8.90	
*MdNPF2.1*	MD11G1122200	Chr11:11243604-11246153	1656	552	60.36	8.14	
*MdNPF2.2*	MD11G1121600	Chr11:11225429-11227624	1404	468	51.09	8.99	
*MdNPF2.3*	MD07G1180500	Chr07:25970318-25972596	1539	513	56.69	9.27	
*MdNPF2.4*	MD01G1112600	Chr01:22682711-22685617	1662	554	60.53	9.25	
*MdNPF2.5*	MD11G1122500	Chr11:11284715-11290091	1698	566	61.85	8.86	MG021338
*MdNPF2.6*	MD03G1108900	Chr03:9496734-9500175	1812	604	66.37	8.73	MG021345
*MdNPF2.7*	MD03G1108700	Chr03:9467766-9470916	1683	561	61.53	8.96	
*MdNPF2.8*	MD06G1186800	Chr06:32435102-32437373	1809	603	67.17	9.61	
*MdNPF2.9*	MD14G1193200	Chr14:28393718-28396166	1866	622	69.34	8.88	
*MdNPF2.10*	MD04G1137500	Chr04:22509802-22511767	1764	588	64.55	8.76	
*MdNPF2.11*	MD14G1193100	Chr14:28376948-28379737	1746	482	64.43	9.00	MG021332
*MdNPF2.12*	MD06G1186500	Chr06:32427308-32429277	1299	433	48.04	8.86	
*MdNPF2.13*	MD16G1080100	Chr16:5611393-5619434	1845	615	68.20	8.44	
*MdNPF2.14*	MD16G1079900	Chr16:5602664-5605755	1818	606	66.73	8.14	
*MdNPF2.15*	MD16G1080000	Chr16:5608051-5610469	1737	579	63.71	7.82	
*MdNPF3.1*	MD13G1043200	Chr13:3030935-3034949	1740	580	64.10	8.61	MG021337
*MdNPF3.2*	MD13G1043100	Chr13:3013976-3017053	1776	592	65.47	8.61	
*MdNPF3.3*	MD16G1044000	Chr16:3127680-3131016	1743	581	64.24	8.34	
*MdNPF4.1*	MD05G1164400	Chr05:29316397-29322734	1308	436	48.37	7.39	
*MdNPF4.2*	MD10G1153900	Chr10:24150970-24153718	1605	535	59.31	9.33	
*MdNPF4.3*	MD10G1154000	Chr10:24171779-24174663	1602	534	59.02	8.26	
*MdNPF4.4*	MD08G1248200	Chr08:31226687-31231330	1842	614	67.84	8.30	MG021342
*MdNPF4.5*	MD15G1443100	Chr15:54322869-54325021	1482	494	54.88	8.96	
*MdNPF4.6*	MD05G1000900	Chr05:293868-298577	1845	615	68.08	8.54	
*MdNPF4.7*	MD13G1079300	Chr13:5562578-5567885	1761	587	64.77	8.58	
*MdNPF4.8*	MD16G1079100	Chr16:5547983-5553360	1761	587	64.85	8.70	
*MdNPF4.9*	MD14G1194100	Chr14:28528146-28530941	1758	586	64.70	8.26	
*MdNPF4.10*	MD08G1040500	Chr08:2990042-2993173	1668	556	61.30	8.22	
*MdNPF4.11*	MD04G1184500	Chr04:27539356-27541647	1836	612	68.06	9.15	
*MdNPF4.12*	MD12G1197700	Chr12:27888187-27890493	1653	551	61.38	8.67	
*MdNPF4.13*	MD10G1271800	Chr10:36356975-36363499	1779	593	66.11	8.08	
*MdNPF4.14*	MD05G1293900	Chr05:42659455-42665121	1809	603	67.05	8.58	
*MdNPF5.1*	MD07G1230600	Chr07:30498119-30501840	1776	592	66.07	8.76	MG021340
*MdNPF5.2*	MD05G1192100	Chr05:32036536-32040692	1809	603	67.23	9.30	
*MdNPF5.3*	MD16G1224200	Chr16:22556239-22559806	1644	548	61.45	8.57	
*MdNPF5.4*	MD13G1218900	Chr13:21148526-21157623	1806	602	67.12	9.36	
*MdNPF5.5*	MD05G1342600	Chr05:46297101-46299829	1779	593	66.26	9.22	
*MdNPF5.6*	MD07G1039100	Chr07:3293256-3295279	873	291	32.36	9.39	
*MdNPF5.7*	MD07G1038900	Chr07:3272734-3276872	1695	565	62.35	9.08	
*MdNPF5.8*	MD07G1038800	Chr07:3237655-3247764	1731	577	63.70	8.82	
*MdNPF5.9*	MD07G1038600	Chr07:3176293-3185619	1695	565	62.63	9.01	
*MdNPF5.10*	MD07G1039600	Chr07:3353966-3358684	1731	577	63.97	8.71	
*MdNPF5.11*	MD04G1148300	Chr04:23666705-23668537	1635	545	60.03	6.25	
*MdNPF5.12*	MD04G1138500	Chr04:22623404-22627516	1770	590	65.59	8.62	
*MdNPF5.13*	MD12G1153900	Chr12:23405312-23409371	1770	590	65.19	8.93	MG021339
*MdNPF5.14*	MD07G1205700	Chr07:28355985-28360202	1794	598	67.03	8.77	MG021344
*MdNPF5.15*	MD01G1141500	Chr01:25051572-25054193	1785	595	66.39	9.24	
*MdNPF5.16*	MD17G1041000	Chr17:2979487-2982673	1632	544	60.48	8.35	MG021336
*MdNPF5.17*	MD09G1040700	Chr09:2607173-2618823	1581	527	58.64	8.79	
*MdNPF5.18*	MD08G1218300	Chr08:28077601-28080578	1080	360	40.06	5.25	
*MdNPF5.19*	MD15G1406700	Chr15:50693334-50695520	1317	439	48.70	6.36	MG021331
*MdNPF5.20*	MD15G1406500	Chr15:50682947-50684829	1377	459	50.92	5.76	
*MdNPF5.21*	MD07G1039200	Chr07:3295281-3306861	825	275	29.88	8.94	
*MdNPF6.1*	MD08G1022500	Chr08:1648693-1654146	1551	517	57.45	9.00	
*MdNPF6.2*	MD15G1019900	Chr15:1155213-1159411	1869	623	69.53	8.46	
*MdNPF6.3*	MD16G1142100	Chr16:10938991-10941873	1914	638	70.17	8.56	MG021341
*MdNPF6.4*	MD13G1131800	Chr13:10003867-10006664	1914	638	70.08	7.70	
*MdNPF6.5*	MD15G1173800	Chr15:13572779-13576346	1746	582	63.62	9.30	MG021346
*MdNPF6.6*	MD04G1086400	Chr04:12553185-12555016	1011	337	36.83	8.33	
*MdNPF6.7*	MD17G1103000	Chr17:8745481-8748650	1773	591	65.11	9.24	MG021333
*MdNPF7.1*	MD11G1017300	Chr11:1392309-1395225	1866	622	67.81	5.82	
*MdNPF7.2*	MD03G1016700	Chr03:1321170-1324060	2022	674	73.81	7.30	
*MdNPF7.3*	MD03G1016400	Chr03:1307373-1311107	1782	594	65.21	6.59	
*MdNPF7.4*	MD07G1082700	Chr07:8103711-8110134	1815	605	67.04	7.80	
*MdNPF7.5*	MD02G1228800	Chr02:27110155-27115346	1815	605	67.22	7.64	
*MdNPF7.6*	MD06G1029400	Chr06:3504379-3509435	1791	597	66.37	7.89	
*MdNPF7.7*	MD16G1277800	Chr16:37688341-37693389	1788	596	66.44	6.71	
*MdNPF8.1*	MD12G1160700	Chr12:24039618-24042029	1707	569	63.32	8.60	MG021334
*MdNPF8.2*	MD04G1147500	Chr04:23563075-23568217	2061	687	76.60	8.76	
*MdNPF8.3*	MD11G1081100	Chr11:6913320-6915864	1728	576	63.96	8.11	
*MdNPF8.4*	MD11G1081200	Chr11:6931888-6934707	1749	583	64.77	8.62	
*MdNPF8.5*	MD16G1010600	Chr16:814341-817396	1758	586	64.39	7.18	

^a^ Gene ID in apple genome (https://www.rosaceae.org/organism/Malus/x-domestica).

## Supplementary Materials

Supplementary Materials can be found at http://www.mdpi.com/1422-0067/19/9/2761/s1.
