# Supplementary material for: Genome-Wide Identification and Analysis of Apple NITRATE TRANSPORTER 1/PEPTIDE TRANSPORTER Family (NPF) Genes Reveals MdNPF6.5 Confers High Capacity for Nitrogen Uptake under Low-Nitrogen Conditions
Source: Int J Mol Sci. 2018 Sep 14;19(9):2761. doi: 10.3390/ijms19092761 (PMC6164405; doi:10.3390/ijms19092761)
Supplement: Supplementary file 1 [file ijms-19-02761-s001.pdf]

Supplementary Materials, Table S1: The 16 deleted NPF gene sequences of apple.

Gene ID	Sequence
MD05G1164700	ATGATGAGGAAGTTTGTTCTTAGTTTTTTGTTTGCAGTTGTTGGGGTGCTGATCAACATGGTTTTC<br>TTATCCAATGCCATTGACTTCGTCTCATACTTCTGGTTGTCAATGCATTACTCACCTGCTACATCA<br>GCAAATATGGTGACCAATTTCATGGGAACCTCTTTTCTGCTCTCCATCGTGGGAGGATTTATTCG<br>TGACTCACTCCTCACAAGCTTCACAACCTTCATCATCTTCTGTGCAATAAACTTAGCGGGAGTA<br>ATCTTGCTGGCAATCCAAGCTCAATTCCCACATTTACAACCAGCTGTGAAAGTGAAGCCTTCTCT<br>GTCTCAAACAACCATTCTATAA
MD13G1080000	ATGAAAATGGATCGTCACATTGGACTGAATTTCGGAATCCCTGCTGCCTCGGCGAAAATGATGT<br>CATTATGACGCTCTTGATTTCCCTCACGTTGAACAACCGTGTCTTACAACGAGCCCTAACAAAGT<br>TCACTAAATGTGAAGGTGGGATCCCACATTTAGTGAGCAACAAAGGAGAACTTTGGCTACATC<br>ACAGTGCTTCATCGAACGGGGTTGTAGCCACGTCGGTTTTTTGGCTATTTCCTCAGCAAATGTTG<br>CTCGGACTTTGCGAGATGTTTGGCGTCGTTGGGCTCCTTGAATTTTACAACCAAGAGTTTTCTGA<br>GAAGATGAGAAGTATTGGGAATTCTCTGCCCTATCTTTGCTTGGCCGGGGCTACTTATCTGAGTA<br>CTGTGGCGGTGTGCATTCTGCGTAGTGTTACAGGAAGGCATGGTCAGCCCAACTGGTTGGACAA<br>TGATATCAATGCAGGATTGGACTATTTTTTACCTTTTTGTCTTGTAAAATTATCACGTGAATATTT<br>TTAG
MD13G1080300	ATGGCGATGATGGCTGAAGGCAAGAAGCAGCAGCAATCAAGCCACTGCCTCTACGTTACAAAG<br>TGGTTTCAGAAAAGCACTGCCTCAGAGCTGTCACACAAATTTCCTTTAAGCCCAGATAAAGAAC<br>CAGTTGATAGAAAACGTGATGTCACAAGAAAACCTGGTGGTTGGAAGGCCATGCCTTTCATCTT<br>AGGAAATGATACGTTCGAAAGGTTGGCTGCTTTTGGGTTGCTGGCAAACTTTATGGTGTACTTGA<br>CGAGGATGCTACACCTAGACCAGGTTTCAGCTTCCAACATTATCAACATCTGGTCTGGTTTGAC<br>CAACTTCGCTCCTCTCCTCGGTGCCTTCATCTCCGACGCCTATATCGGCCGCTTTCGGACTATTGC<br>TTTTGCGTCCATTTCTTCTCTCATGAGGTGGGAGAGCGCCGGTGATGATCTTGTAGTTGGCTTTGT<br>TGTCTTGTTGTACGCAAAATCTCAGAAACTCTTTGAAAAGGTACCCAGTGAGTTGATATATGGA<br>TTGGAAAGGGGAATGGCGACAATTACTTTAACAGCGTGGCTGCCGCAGCTGCATCCTCCGCCAT<br>GCAGCCCACAAAAGCAAGCACTGAGTCAGTGCACCGGTCCAACCCGAGCTCAACTCGGTCTCC<br>TGCTACTGGGTCTAGGGTTTCTGTCCATAGGCTCAGGTGGCATCAGGCCATGCAGCATCCCATTT<br>GGCGTTGACCAGTTTGACCAAACCACCGAAGAAGGAAAGAAAGGAGTGAAAAGCTTCTTCAA<br>CTGGTACTATGCTACGTTCACAGTGGTCTTGTTGATCACCCAAACCGTAGTGGTTTACATTCAAG<br>ACAAAGTCAGCTGGAGTTTAGGGTTTGGGATCCCAACGGTGCTAATGGCTTGCTCTATCCTGCTT<br>CTGCTTATCGGGTCTAGGGTTTACGTGCATGCAAAGCCACAGGGCAGTATATTCTCTGCGATTGC<br>ACAAGTACTTGTTGCTGCTCATAAAAAACGCCACGTTAAGCTTGCGGAAGAAGCAGAAATTAT<br>AGAAGTTGACAAATTCTATGATCCTCGAATATTCAAGGGGCATTACGCCTCTAAGCTCCCTCTC<br>ACAAACCAGCTCAGGTTCTTGAACAAGGCTGCTGTAATATTGGATAACGATCTGAAGCCTGATG<br>GTTCTCCAGTCGATAACTGGAGGCTGTGCAGCGTGCAACAAGTGGAAGAACTTAAATGCGTATT<br>AAAAACAATCCCGATATGGTCAACGGCCATAGTTAGCCTGACCGCCATGATACAGCAGGGCAC<br>GTTTACTGTGTCCCAAGCTCTAAAAATGGACCGTCATCTCGGCGCTAACTTCGAAGTCCCAGCT<br>GGGTCCATCAGCGTCATCTCCTTTCTCACCATTGGCCTCTGGCTCCCAATCTACGACCGGATTAT<br>AGTCCCGTCCCTCAGAAAATTTACAAAGCTCGAAGGTGGGATTACAGTCCTGCAGAGATGTGG<br>AATCGGGATTATATTCTCCGTCCTGTCCATGGCAGTGGCCGGAGTAATCGAAAAGGAGAGAAG<br>GGATTTTGCAAATTTGCACCCTGACGCACATGTGTCATTCCTCTGGCTAGCCCCGCAACTTATTC<br>TGATGGGGCTCTGTGAGGCGTTCAATATTATAGGTCTGCTGGAGTTTTTCAATCGGGAGTTCCCC

	GACCACATGCGAAGCGTTGGAAATGCCCTTGTGTCTTGCTCCTTTGCCGGTGCGAGCTACTTGAG<br>CAGTTTCACGGTGACTGTGCTGCACCATGTGACTGGGACAGAGAAGCACCCGGATTGGTTGACC<br>AATGACCTCAATGCTGGGAGATTGGACTACTTCTACTTCCTATTGGCTGGGATTGGGGTTCTGAC<br>TTTTGCCTACTTTCTGTTTTGTGCGCAACGTTATCATTACAAGGCCACAAATGTGAACGTTGAAG<br>AGAAGCCATATGATACTGATCAGGTGCAGTTAGGTTCAACAAATGCTTAA
MD06G1186600	ATGGAGAAGAAAGAGAAAGAGAGCATGGAGAAGAATGAGAGAGAGAGCATGGAGAASAATG<br>AGAGAGAGAGCATGGAGAARAATGRGAGAGMGAGCATGGAGAARAAWGAGAGAGMGAGCA<br>TGGAGAAGAATGAGAGAGAGAGCATGGAGAAGAATGAGAGAGGTTCTACGGATGAAGAACC<br>AGTTATATACAGAGGATGGAAAGTAATGCCATTTGTCATAGGAAATGAAACGTTTGAGAAGCT<br>GGGAACCACTGGGACCTTGGCCAACCTCTTGGTCTATCTCACTAGTGTATTCAACATGAAGAGA<br>AATACAGCTGCGACTCTTGTTACCACCTTCAATGGCACCACCAACTTTGCAACCTTGCTCGGAG<br>CTTTCGCTTCCGACACTTATTTTGGCCGTTACAAGACGTTAGGATTTTCGACAATCGCTTCTTTTA<br>TGGTACTCACAAAACTAACATTTTCTACTTATTAG
MD14G1192700	ATGAACCCGGATGGATCAGCTGCTGATCCGTGGGGACTTTTTAGCATGCAGCAAGTGGAAGAA<br>GTGAAATGCCTGCTGAGAGTGTTGCCAATATGGGCAGCAGCCCTCGTATTCCATATTCCCATAG<br>TCCAGCAGCAAACCTTTGTTGTCTTCCAAGCCCTTCAATCCAATCGACGCCTCGGAAAAACCAG<br>CTTCCAAATCTACGACCGCCTTCTTGTCCGATTCCTCCAAAGGCTCAAGGGAAAAGAAGGCGGC<br>ATCACATTGYTGCAAAGAATTGGAATCGGCATTTTTCTCTCTGYGATCGCCATGCTAGTATCTGG<br>ATTTGTAGGACAMYGAAGGACTATAGCGCTAACAAAACCAATTCCAGGAATCCAGACAAGGG<br>GTGACATTTCATCCATGTCCGGCTTTTGGCTGGTCCCTCAGCTCACACTAACCRGGCTTGCTGAA<br>GCGTTCACCTCCGTTGGCCAAGKCGAGTTCTACTACAAGCAATTCCCGGAAAACATGAGAAGC<br>ATTGGAGGGTCTATCTACTTTTGTRGACTGGCTRGTTCTAGTTACTTGAGTAGTGCGTTAATCGTC<br>ATAGTTCACCGGAMTACTGAGAGGGCTGAGACCGGGAATTGGCTGCCGGAAGATCTCAACAA<br>GGGGAAGCTGGATCACTACTATTACTTGATTGCTGCTCTTGGAGCCATTAGTTTGGGATAA
MD12G1152300	ATGGAGCATGCAGCAGAGTCTTCATCTGAAGGGCTAGAGAGTCAATTCCTTCCTCCGCAAAGAC<br>CGGTTAGAGGATGGAGAGCCGTCAGATACATTCTCGGGAACGAGACTTTTGAGAAGTTGGCTTC<br>CATGAGTTTGATTGCTAATCTGGTGGTGTATCTGCACACAAAGTACCACTTGGACAACGTGGTTT<br>CAGCTAATGTGTTTAATATATGGTCTGGCTCATGCAACATTGCACCATTGTTTGGTGCTTTTCTTG<br>CTGATACTTATTTGGGAAAGTCTTATACTCTTCTTTTCAGCTCGATAGCATCACTTCTGGGAATGG<br>GGACCCTGACCTTAACTGCATGCATACATAAATTAACTCCGTTTGCCTGCGCTGCGCAATGCCA<br>ACAGCCGAGTACTTGGCAGATAGCGATCCTGTATTCGGGTCTCGGATTGCTTGTTGTTGGATYCG<br>GAGGCCTTAGACCATGCAACATTGCCTTTGGAGCTGATCAGTTTGACACCACAACGGAGAAGG<br>GAAGAGAAGAGGCACCTGCTTTCTCAGCCTTGTCAGCACCTGTCACATGCACACTCATCTTTGC<br>GGAAATTACGAGCAATCTGTCGAAGATTTCTGGTGACGTAGAAAGCACGGGAATCTTACTGTTC<br>AATCATCCACTTTCCACACGCAACATCAGCTCATGGGTACCACAGATAACTTTGCCAAAGATCT<br>CTGACAAAGTTTAG
MD03G1109300	ATGGGCTTAGTCGTGGGCACCTCAACATGGTTGGGTGCGGGGTGGAAACAAAACATGCCAAAA<br>ATTTGGCTTGGGCAGAGGAAAGGGACAGGCCAGCAGCATCGGGCTGAAAAAAAAAGGAAGGT<br>GGCCCATAAGACAGGGCGGTCCGAGAAGCAGGAGCCCAGCATGGCAGTTGCAGGCTGCTGGG<br>AGAAGCCCAAGAAGTTGGGTTTGGGCTCAGCTGAGGCAACAAGCCCAAACATGGGGATGCAG<br>GTCGAAACACCAAACGGTGTCGTTTTGGAGCAGTGGTCAGAGCTGCAGGCTCGGCTTCAGGCC<br>AAGCCAGGGGCTTTGGGAGGCTTGACACTTGCAGCTGGGGGATGGATCTCAAATTTGATTGTRT<br>TTCTAATTAAAGAGTTCAATATAAACAGCATTGATGCAGCCCAGATCGCAAACGTGGTTAACGG<br>TTCTTTCAGTTTTTTCCCAATCATTGGAGCAATCATAGCTGACTCTTTCTTTGGCTCATTCTCCGTT

	ATCTCAATATCCTCATGCATTTCCTTGCTGGGCATCGTTCTCTTAGTTTAACCGCAACGCTGAAT<br>TCGTTGAAGCCTCAACCGACCTCGGCATTACAGTATGCAGTCTTGTACACCGGCATAACTGTCG<br>CATCTCTTGGCTTGGGAGGTACCCGCTATACCTTGGCAACACTGGGAGCCAATCAATTTGATAA<br>GCCTAAGCATCAAGCAAGTTTCTTCAACTGGTTCTTCTTCACTCTATACTCTATTACAGTGGTAG<br>CCCTAACAGTCATTGTCTACATTGAAGACAACGTCGGTTTTAAATGGGGGTTTGGCCTTTGTGTC<br>ATCGCCAACCTAATCGGAATGGCCATTTTCTTCTCTGGAAACCGTTTCTATAAATTTGATAAGCC<br>ACACGGGAGTCCATTTGTTGGTTTAGGTCGTGTTATTGTTGCCTCTGTTCGGAAAACTAGTCTCC<br>AGCTATCATCTGAAAGAAAGGATTATTATTATGGACATGATGGAGTGACACATGACGGAGTGA<br>CAAACAAGGTGGCTGCAGCAACACTTAGCAAGAGTTTCAGATTCCTTAACCGTGCAGCACAGA<br>AAATTGAAGGAGACATGAAATCAGATGGCTCAATTGCGAAACCATGGAGCTTATGCACAACGC<br>AACAGGTGGAAGATTTCAAGACCGTTATAAGAATTTTTCCACTATGGTCTACAGCTTTATTCTTA<br>GCTACCCCAATAGCAATCCAATCCAGCATGTCAGTCCTGCAGGCTCTAACCATGGACCGTCACA<br>TTGGCCCCCATTTTACATTGCCATCTAGTTCTGTTATAGTCGTTGTTTTATTCTCCACATCCATTTT<br>TCTAACCCTAATTGATAGGTTCTTATGCCCCATGTGGCAGAAGTTCACTGGTCAGTCTCTATCGC<br>CCCTCCAGCGTGTAGGAGTAGGACACATCTTAACCATTCTCAGCATGGCTGTTTCAGCAGTGAT<br>GGAGTCCAAAAGGCTCAAAATAGTGCAAGAACACCACCTCCAAGACCAGCCCGGTACTATTGT<br>TCCAATGTCGATCTTTTGGCTTTTCCCACAGTTAGTTTTGGTAGGCATTGGAGAAGCATTTCATTT<br>TCCAGGACAAGTTGCATTGTATTATCAAGAATTTCCCATGTCGCTGCGAAGCACATCGACGGCC<br>ATGATCTCATTG<br>ATTATTGGGATTGCATATTATGTAAGCACAGGTGTGGTTAATGTGGTCCAAAGGGTTACTGGAT<br>GGTTACCAAACAATCTAAACAATGGGAAGCTGGACAATGTTTATTGGATGCTAGCTGTGCTGGG<br>GGTGATAAACTTTGGCTATTATTTGGTGTGTASTAAGTTGTACAAGTATCAAAATGTAAAGGGTG<br>CAGAAGGTAGTCCTGACCCAGATAGCTAG
MD11G1122300	ATGGTGGATGATGGTGGAGCTTTAATGTGCTTGACACTTGCTGCTGGAGGGTGGCAAGCAAATC<br>TGATTGTGTATCTGATTCTGGAGTTTAACTTGAAGAGCATTGATGCTACTCAGGTGTCAAATGTT<br>GTCAATGGTTGCACTAGTTTGTTTCCTATCATTGGAGCAATTGTTGCAGACTCTTTCTTTGGCTGT<br>TTCCCTGTCATCCTGATCTCGTCTTGTATCTCTTTTCTGCTCTGGTTTTTCTTTGCCATGTATACAG<br>CGACTGTTACAAGTTCTACTGYCGTTGTCTATATTGAGGAYAGTGTCAGTTGGGGGTTGGGATTT<br>GGCCTAWCGGCTCTTGTTAATWTGATTGGTCTGGTCATATTCTCATCTGGAATCCATTTCTATCR<br>TCGTGATAAGCCACAAGGTAGCCCATTYGTGAATATAGCTCGTGTTATTGTTGCTAGTATCCGGA<br>AATGGAATGTTAAGCTCTCATCCAGAAGGGAGGATTACCAGTACCGCAATGATGGAGAGGCCA<br>AGACGACTCCTGCAGCACCTAAAAAGAGTTTCATTACTATTTCTGTAGGAAACAGAGTGCCATG<br>CACAATCAAGGAAATAATGATGTGCAGCCCTGGAACAGAAGGGGACCTGAAGCCTGACGGCT<br>CAATTGCAAGGCCATGGAGGTTGTGCACACTCCAGCAATTGGTTTTAGTGGGCTGCGGTGAAGC<br>ATTTCATTTCCGGGGCAAATTCAGTTTTGCTATCAAGAATTTCCAGCATCACTTCATACCACGGC<br>GACTGCAATGGTTGCGGCGTCTTACTTGA
MD15G1406600	ATGGCAACTTCTACCCGCCAATCCCCGGACCGGGACACCCAAACCCCYCTGCTAGACGACGGC<br>GTCGAGGGCACCGTTGACCACAAAGGCCKCCCCGTTCACAGATSGAGATCAGGCGGCTGGCGT<br>TCTGCTCGGTTCATAATCGGGGTGGAGCTGGCGGAGAGGTTTGCCTACTATGGAATCAGCTCCA<br>ACCTCATCACGTTTCTGACGGGGCCTTTGGGGCAGTCCACCGTCACAGCGGCTGAGAACGTCAA<br>CATATGGTCCGGAACGGCGTCGTTGCTCCCTTTACTCGGAGCCTTTGTCGCTGATTCTTTCCTTGG<br>GCGCTACCGCACTATTGTTTTTGCTTCCCTACTCTACATTTTGGTACGTCTCCCTCTCTCTACAATT<br>TTGGCACGTGACTATGTTGTTGGAGTTTTTAGCTCCTTTAAGTCCCACATCGGGGAACTCAAGGA<br>AACTTGA

MD12G1154200	ATGGGAGGCTTCTGTRCCACACGAGGAAGCTCAGCTGCCAATAGGAATCAGATTACCAAGATT<br>GATAATTATAGTGTAAAAAGACTCATCCTTCTGAATACAAAGCAAATGAAAATCACATATCCG<br>CAAAAAGTTCTRMACATTGATGAGAAGAACATGTTCAGAGGATTTCTTGACAAGGCGGTGATC<br>ATAAGCGCACAAAACTTGGCTGAGAATCCAAGCCCTTGGAGACTAGCAACTGTGACCAAGGTG<br>GAGAAGATGAAATTTGTTCTCAACTTGATCCCAATTTGGCTAGCGACTCTACCATTTGGAGTGTG<br>TGTGGCACAATCCTCCACATACTTCATCAAACAAGGTGCCACCATGAACAGAGAGGTTGCAAA<br>TGGTTTCGAGGTCCCCCCTGTCTCAATCTTCGCACTCGCAGCCATTGGAATTATCGCCTCCGTCA<br>CGATATAA
MD02G1288100	ATGTCGCACTATGGGCTAATCGTAATAATTTTGTATAGAACTCGTCATTCACAAGAGTCCAAGTT<br>TAGACTTCCAGCTTACAAGCACTATCAAGTGGAGAGAATTGTTGAGGTGGCAGAGAGATTTGTT<br>TACCTTGGCATGTCTGGTAACCTCGTCACATACCTCACAAATGAACTCCATGAGCCAATCCCCA<br>TGGCTACAAAGAATGTCAACACTTGGGTTGGAGTCTCCTATCTCCTCACAGTAATTGGAGCCTTC<br>ATAGCCGATGCCTATCTTGGTCGATTCAAAACCATCCTCGTCTCGTCTATCATTTACTGCTTCGC<br>AACGGTGGTGTTGAACATTACAGTTTCAGTAATTCCTCTGCATTATCACAAAGCAATGTTCTTTG<br>TAGCACTTTACATATTGTCAATTGGGCTAGCTGGCCACAGACCGTGTGCACAAACTTTTGCGGTT<br>GACCAGTTTTGTGAGGACTCGCCAGAGGAAAAGAAGGCGAAGAGTTCGTTCTTCAACTGGTGG<br>GTTTGGAATACTGACAGGGGCAATGGTAGTGGCCTTGGCTCTGTTCTTGTTCGGTTACAAGAAGT<br>TCAGATGGCCGGGACCCTTAGGCAGCCCCTTCACCATGGTGGCTCAGGTGTTTGTGGCGGCAGC<br>GAGGAAGCGGCACCTCGATGGAACATCAACTGGTTTTGCCCGAAAAATCACAGACATTCCTCT<br>GGCATTACCATCCTACAACGAATCGGCATTGGCCTATTTCTATCCATAATCACCATGGTCGTGTC<br>AGGTTTGGTAGAAGCGAAAAGAGTTAGCGTTGTAAAAGATCACAACCTCTTGGACAACCCGAA<br>AGCAGTAGTGCCAATGAGATTGTGGTGGTTAATTCCACAGTACATGGTATGCGATTTATCTGATT<br>TATTTGCATTGGTCGGAATCCAAGAATTCTTTTACGATCAAATGCCGGAGGAAATGAGAAGCAT<br>GGGAGCGGCAGCATACCTTAATGTTAATGGTGTCGGAAGCTTCATAAGCAGCGGTATTATAACC<br>CTGGTGCAAGCGATTAGCTCAAAGCATGGTGAAAAATGGCTTAATGACAACATCAATCGTGCC<br>CACCTCAATTACTTTTATTGGGTAATTGCAGCTTTGAGCACTTTGAACTTGTGCGTTCATCTGTGG<br>ATTGCTAAACGTTTTGTATACAAAAAGCTTGCAGCAAAAATTTCGATTTCGATTTCGAATATGTT<br>ATCTGTAACTGTGAGGGCATCTCCATCTCCATCTGATTCTGTGTTTGATTTTAGACTTGGTGGAGG<br>ATCAATCGGTGGGAGATATGAGGAACCCAGTTATGAATACCGAAAAAAACGCAATCGTGATGG<br>TAGCCTCAAAGAGTTAGGGTTAGGGTTAGGGTTTTCCACCACTTGTACAGCACCTGCATATGAA<br>TACCGAAAAAAACGAAATCGTGATGGTAGCCTCAAAGGGTTAGGGTTTGAAACTACTTGTGCT<br>GAGGCTGAGACCACTGTGGAAAAGAAGACAGCTGATGAAGAAATCGCCGTTCCTGCTGATGAA<br>GATCAGATTTGGTGCGTGTTATGTGGCGAATGTTTTGACGATTTCTATAGTCATGAGACCAAGGG<br>ATGGATGTATAAAGGTGCTGTGTACTTGAATGCACCTGAGGGATCAACAGGGAACACGGATAG<br>AAATGACACGCTGCGGGATAAGAAGGCGAAGAGCTCATTTTTCAACTGGTGGTTCTTGGGGGTC<br>GTAATTGGTGCCTCTGCTGCCATTTTGGTAGTCATATATGTACAGGATTATGTTGGGTGGGCGGC<br>AGGGTTTGGAATATTGACAGGGGCAATGGCGGTCGCATTGGTTCTGTTCCTGTTCGGTTACAAG<br>AAGTACAGAAGGCAGGGACCCTTAGGCAGCCCTTTCACCTCGGTGGCTCAGGTGTTTGTGGCGG<br>CAGCGAGGAAGTGGCGCGTCAATGGAACACCCACTGGTGGTGTTGTGCACAGTGATGACAAGA<br>GTCAACACGAGACTAGGACTTTGGCGCATACCAGTCAGTTCAGATGCTTGGACAAGGCAATGA<br>TCGTTGACGACCATGATGCTTCATGCA<br>TGACCAGAAATCCATGGAGATTATGCTCACAAAATCAAGTAGAACAAGTAAAGCTCATCCTAC<br>GCCTGATTCCCGTATGGATGTGTTGCTTAATGTTTGCTGTAGTCCAGTCCTTTTTCCAGACCACCT<br>TCACCAAGCAAGGAAGCACAATGGTCCGGTCAATTGGTTCAAACTTTGTGCATCCCGAAGCATC

	ACTTCAAATCTTCGTTAGCCTGACAGTTATAGTTGTCATTCCAATCTATGATCGTGTTTTTGTCCC<br>AACTGCCCGAAAATTCACGGGACAGTTTGGTAGAAGCAAAAGGGTTAGCATTGCAAGAGAAC<br>AAAACCTCTTGGACAACCCGAAAACAATAGTGCCAATGAGAGTGTGGTGGTTAATTCCACAGT<br>ACATGGAAATGAGAAGCATGGGAGCAGCAGCATACCTCAGCGTTATAGGTGCTGGAAACTTCG<br>TAAGCATTAGTATAATAACTGTGGTGCAAGTAATTAGCTCAAAGCATGGCGAAACATGGCTTAG<br>TGACAATATCAATCGTGCCCACCTCGATTGCTTCTATTGGAAAATTAACACAGCAAAAGCAAGA<br>ATGGGTACTGGTGAATGGTTCAGACGAACGGCTGGGACTGGGTCARGTTGCAGCRATGCGGGT<br>GTGAGTCGGGTTGATCTGATATGGTGCATGTATGGTTCTGTCGGTGCTGGTGCTAGGCWTATGC<br>GAGCAARTGTAGCGAGGATGGAGAGATTTAGGACTGAGTTGCAGGTGATGGGTCGGCGCCGGA<br>GATTCAAGTGGGTCTGGCAACGGGTGGTGGTGCGGCTGGTGTTGGATACGAACGGCAGATGTG<br>CTGGGTTTCTAGTTGCAGACCTGGTACGAAGGYTGATGGCTGGGTGA
MD02G1287700	ATGTCGCACTATGGGCTAATCGTAATAATTTTGTATAGAACTCGTCATTCACAAGAGTCCAAGTT<br>TAGACTTCCAGCTTACAAGCACTATCAAGTGGAGAGAATTGTTGAGGTGGCAGAGAGATTTGTT<br>TACCTTGGCATGTCTGGTAACCTCGTCACATACCTCACAAATGAACTCCATGAGCCAATCCCCA<br>TGGCTACAAAGAATGTCAACACTTGGGTTGGAGTCTCCTATCTCCTCACAGTAATTGGAGCCTTC<br>ATAGCCGATGCCTATCTTGGTCGATTCAAAACCATCCTCGTCTCKTCKATCATTTACTGCTTCGC<br>AACGGTGGTGTTGAACATTACAGTTTCAGTAATTCCTCTGCATTATCASAAAGCAATGTTCTTTG<br>TAGCACTTTACATATTGTCAATTGGGCTAGCTGGCCACAGACCGTGTGCACAAACTTTTGCGGTT<br>GACCAGTTTTGTGAGGACTCGCCAGAGGAAAAGAAGGCGAAGAGTTCGTTCTTCAACTGGTGT<br>GTCGGGTGRGCGGCAGGGTTTGGAATACTGACAGGGGCAATGGTAGTAGACTTGGCTCTGTTCT<br>TGTTCGGTTACAAGAAGTTCAGAAGGCAGGGACCCTTAGGCAGTCCTTTCACCACGGTGGCTCA<br>GGTGTTTATGGCAGCAACGAGGAAGCGGCACCTCGATGAAACATTAACTGGTTTTGGTGTGTAC<br>AGAGATGACAAGAGTCATGAGACTAACAGGACTTTAGCACATACCAGCCAATTCAGATGCTTG<br>GACAAGGCAATGATCATTGACGACCACGATGCTTCAAGCACGACCAGAAATCCATGGAGATTA<br>CGCTCTCAAACTCAAGTTGAACAATCCAATCCTTTGTTCAGACCTTCTTCACCAAGCAAGGAAG<br>CACAATGGTCCGCTCAATTGGTTCAAACTTCAAGCTTCCGCAAGCATCACTTCAAATCTTTATTT<br>TTCTCACGCCCGAAAAATCACGGGCATTCCTTTGGCATTACCATCCTACAACGAATCGGCATTG<br>GCCTATTTCTATCCATAATCACCATGGTCGTGTCAGGTTTGGTAGGAGTGAAAAGAGTTAGCGTT<br>GCAAAAGATCACAACCTCTTTGACAACCCGAAAGCAGTAGTGCCAGAGAGAGTGTGGTGGTTA<br>ATTCCACAGTACATGGTATGCGGTTTATCTGATTTATTTGCATTGGTCGGAATCCAAGAATTCTTT<br>TACGATCAAATGCCGGAGGAAATGAGAAGCATGGGAGCAGCAGCATACCTTAATGTTAATGGT<br>GTCGGAAGCTTCATAAGCAGCGGTATAATAACCATGGTGCAAGCGATTAGCTCAAAGCATGCT<br>TTGAGCACTTTGAACTTGTGCGTTTATCTGTGGATTGCTAAACGTTTTGTATACAAAAAGTTTGC<br>AGCAGAAGCAACCAACGAGGAGAAAGAACGGACTTAA
MD00G1201300	ATGAGTGATAGTACTACCCTCCCAGATACACAAGGAAAAATCACCCTCCCAGATGCATGGGTC<br>TACAAAGGCTGCCCAACCGAGCGGTCAAAAACTGGCGGCTGGACRGCGGCCGCCATGATTCTA<br>GGKATAACTAGCTACCCACCCTGCCTATGTGGAGAGGCATGTGAGCGGCTAACAACGCTAGGA<br>ATTGCTGTGAAYTTGGTGACTTATTTGACGGGCACCACGCACTTGGGCAGTGCTACCGCGGCCA<br>ATACCGTCACCAACTTTCTTGGAACTTCCTTTATGCTCTGCTTGCTCGGCGGTTTTGTGGCAGACA<br>CCTTTCTTGGCAGGTACCTGACAATAGCAATATTTGCCACCATCCAAGCAATGGGTGTGACAAT<br>ATTGACCATCTCAACCACAGTCCCCAGCCTCCAACCCCCCAAATGCACCGCTGGAACCGGCTC<br>GCAGTGCCTCCCGGCCAGTGGGAAGCAGCTGACGATCCTCTACACAGCCCTCTACCTCACCGCC<br>CTCGGCACAGGAGGCCTTAAAATCGAGCGTTTCAGGCATAGGCTCACTCGCTGCAGTCACAGTT<br>CTTGTTTACATCCAAGACAACTTGGGGAGGCAATGGGGTTATGGAATCTGTGTCTGTGCAATTGT

	GCTCGGCCTCATTGTGTTCTTGTCAGGCACACGACGGTACCGTTTTAMGAAACTTGTGGGYAGC<br>CCTCTGACCCAAATTGCAGTGGTGYTTGTGAACACGTGGAAGAAGAGGAATATGGAACTGCCG<br>TTGGACTTGACTTTGTTGTACAACGTTGGTGA
MD04G1086300	ATGATCATCAGAATGCTACCAATCTGGGCCACTACCATCATGTTTTGGACAATTTATGCCCAAA<br>TGACCACATTCTCAGTGTCCCAAGCAACCACCATGAACCGCCACATCGGAAAATCGTTCCGAAT<br>TCCGCCGGCATCCCTCACTGCTTTCTTTGTAGGCAGCATTCTGTTAACTGTCCCGGTTTACGACC<br>GGATTATAGTCCCAGTTGCAAGAAACTTACTAAAAAACCCTCAAGGACTAACCCCATTGCAGC<br>GCATAGGGGTTGGTTTGGTATTCTCAATATTTTCCATGGTTGCAGCAGCACTCACAGAAGTCAA<br>GCGTATGAATGCTGCAAGAGCACATGGTTTGACAGGCAATCCAAAGGCTGAGATCCCACTGAG<br>TGTTTTCTGGCTGGTCCCACAGTTCTTCTTTGTGGGGTCAGGAGAAGCCTTCGTGTACATTGGGC<br>AGCTGGACTTCTTCCTCAGGGAGTGCCCGAAAGGAATGAAGACTATGAGCACAGGGCTGTTTCT<br>GAGCACCCTCTCATTAGGGTTTTTCGTGAGCTCACTGTTGGTGAGTTTAGTGCACAAGGTGACAG<br>GGGACAGGAAACCATGGTTTGCAAACAATATCAACCGAGGAAAGCTTTATGATTTCTATTGGCT<br>TTTAGCGCTTTTGAGTGCTTTGAATTTGGTGGTCTATTTGTTTTGTGCCAATTGGTATGTCTACAA<br>GGACAAGAGGCTTGCTGAGGAGGGCATAGAATTGGAGGAAGTAGAGACTTGTGTGCATTAA
MD14G1020200	ATGGCTTGCTTAAAGGTCTGCAAGGAGGGTCAGTTCAAAGAAGATCAAGATGCATGTACTCTTG<br>ATGGTACGGTTGATATGCATGGCCACCCTGCAATCAGAGCAAAATCTGGAAAATGGGTTGCTG<br>GAATCATTATACTTTTGAATCAAGGTTTGGCTACCCTAGCATTCTTCGGAGTCGGGGTGAACTTG<br>GTCTTATACTTAACGAGGGTGTTGCAACAAGACAAAGCGACGGCTGCTAACAACCTCTTTTTGC<br>TGAACCCTAAAGGTTGTGGCAGCAAAGCAAATCCATGTAGATCTCATTCGAGCTGGAAAATTTG<br>GCTGTTCTACCTCTCTATCTACCTAATTGCCCTAGGATATGGTGGGTACCAACCAAACATTGCAA<br>CATTTGGAGCTGATCAGTACGATGAAGAGCACAGCAAGGAAGGGCTTGCGAATGTGGCGTTTTT<br>CAGCTACTTCTATCGGTTTATGAACCTCTGCTCCAAATGCTACAGAGACTTCTGCCTCAAGGAGC<br>AGCAGCAGGCGTCGATCAAATCCACCGTCGAAGCCTCCCTCTCCGCCTCCGCTGCCGATGTCGC<br>TTCTTCCTCTCTCTCCGCTCCTTCTTCCCCTCCTTCAACATCCTTGCCTGCCTCTCCGGCAGCGATT<br>GAGACTATCTGCCAACCTCTGTCCCCGGCCTTGACTTTGTCGGAGGGAGCCGGAGATATAATCG<br>GTGAGCCCGCTGAAGTTGTCCGGGCTCTTGAGGTGGATACGGTGGTGTCGCAGCCGAACCGGTG<br>TACCGTTTGCAGGAAATGCTTCGGGTTGACCGGGTTCAAGTGCAGGTGCGAGACCACGTTCTGA
MD07G1039200	ATGAATATTGCTTCTGGAAAACCCAGTAGGGGTGGCTGGAATGCTGCTATTTTCATCATCTTGGT<br>TGAGGTGGCCCAGCAGTTCGCATTCTATGGGTTGGCAAGTAACCTCATCATGTACCTCACGGAT<br>GTTTTGGACCAGCCCTTAGCCGCGGCGGCTAAGAATGTTAACACCTGGCTTGGTGTCTCATCGGT<br>CTTCCCTGTCCTCGGAGCCTTTCTTGCAGATTCATACTTTGGTCGGTTCAAAACCATAGTCTTCTC<br>CATCACCATCTATTTCCTGGGCATGGTTCTTTTGACTCTGTCCGTCTCAATAATTCCTCCACAAAG<br>TGGCCGAGTAGTGTTCTTTGTGGCGCTGTACATTTTATCGGTGGCTGAAGGTGGGCAGAAGCCA<br>TGCGTGCAAACATTTGCAGCAGACCAGTTCGATGAGAACACGCCTGAAGAGATAAAGGTCAAG<br>AGCTCCTTCTTCAACTGGTGGTACTTGGGGGTGGTATTTGGTGCCACTTCTGCCACACTGGGAGT<br>CGTATATCTCCAGGATAATATTGGGTGGGGATTAGGGTTTGGAATATTGGCAGGAGTTTTGGTG<br>GTGTCACTAGTATCTTTTTTGTTGGGAACGAAGAGGTACAGGAAGCAAATGCCTCCAGGGAGCC<br>CTTTTACTATGGTGGTACAAGTGCTCGTTGCAGCAGCTCGGAAATGGCATGTCAATAAGGCACT<br>CAATAGCGAGTGTGTTTATTATGGAGATGAGGGTGAAGCCCTGCTACAAGGTCAGTCGCAGCCT<br>CTAGTTGTGGCTCGTGCTAATCAACTCAGGTACAAACTTAATATTGCATAA

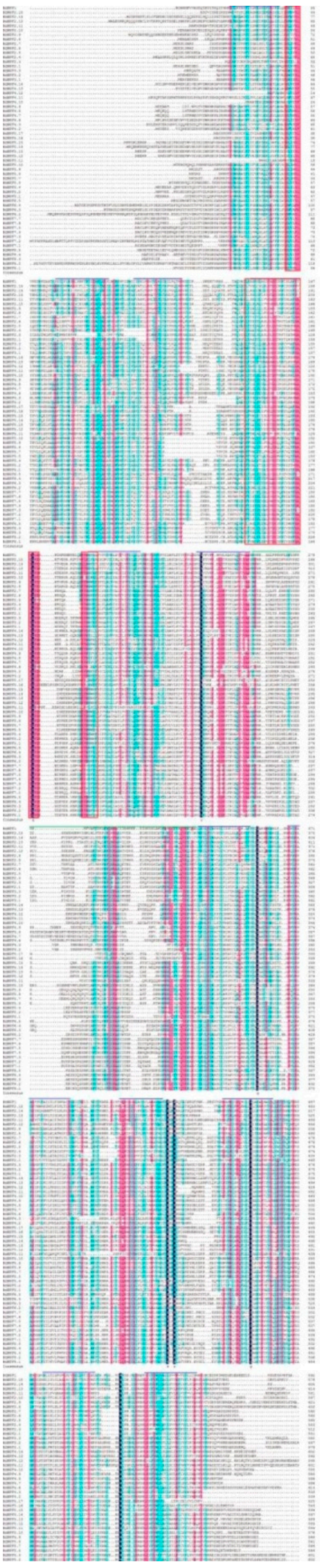

Supplementary Materials, Figure S1: Transmembrane regions and conserved motifs of NPF in apple. Blue lines, TM regions; green line, large hydrophilic loop; red rectangles, three conserved motifs.

Supplementary Materials, Table S2: Analysis of exon lengths for orthologs of *NPF5.1*, *NPF 5.13*, *NPF 5.14*, *NPF 6.3*, and *NPF 8.1* in various plant species.

**Gene**	**Species**	**Exon length (bp)**						
		**Gene name in PLAZA 3.0**	**E1**	**E2**	**E3**	**E4**	**E5**	**E6**
NPF8.1	*Citrullus lanatus*	CL02G19990	109	218	551	835		
	Cucumis melo	CM00084G00310	109	218	551	835		
	Carica papaya	CP00056G00990	106	218	554	829		
	Carica papaya	CP00112G00570	106	218	557	835		
	Citrus sinensis	CS00026G00740	109	218	563	751	39	
	Citrus sinensis	CS00145G00060	106	218	551	832		
	Eucalyptus grandis	EG0002G22800	115	218	551	832		
	Eucalyptus grandis	EG0010G16690	106	218	554	835		
	Eucalyptus grandis	EG0010G16790	106	218	554	835		
	Fragaria vesca	FV3G21860	154	218	554	835		
	Fragaria vesca	FV6G11070	109	218	554	835		
	Fragaria vesca	FV7G18610	154	218	554	798	1627	
	Glycine max	GM11G23370	106	218	560	835		
	Glycine max	GM14G37020	106	218	557	835		
	Glycine max	GM18G07220	106	218	560	835		
	Gossypium raimondii	GR03G12470	106	218	554	835		
	Gossypium raimondii	GR04G00670	106	218	554	853		
	Gossypium raimondii	GR06G18070	109	218	551	832		
	Gossypium raimondii	GR09G09390	71	113	218	554	835	
	Manihot esculenta	ME03150G00520	106	218	554	835		
	Manihotesculenta	ME03651G00110	106	218	551	832		
	Manihot esculenta	ME08270G00110	109	218	554	760	77	16
	Medicago truncatula	MT3G462710	106	218	554	835		
	Prunus persica	PPE_006G00640	136	218	557	835		

	Populus trichocarpa	PT01G24860	106	218	554	835		
	Populus trichocarpa	PT09G04240	106	218	554	835		
	Populus trichocarpa	PT16G11180	103	218	551	838		
	Populus trichocarpa	PT16G11190	103	218	554	832		
	Ricinus communis	RC29751G00030	106	218	557	835		
	Solanum lycopersicum	SL06G076750	229	132	218	554	835	
	Solanum tuberosum	ST06G032510	28	132	218	554	825	
	Solanum tuberosum	ST09G011650	109	218	548	838		
	Theobroma cacao	TC0005G02130	106	218	551	832		
	Theobroma cacao	TC0009G30130	106	218	554	832		
	Vitis vinifera	VV06G02280	106	218	554	832		
NPF5.13	*Citrullus lanatus*	CL04G07660	163	218	554	430	453	
	Citrullus lanatus	CL10G19500	166	218	545	850		
	Cucumis melo	CM00002G01170	151	218	554	436	450	
	Carica papaya	CP00056G00450	154	218	545	874		
	Capsella rubella	CRU_004G19200	145	218	545	844		
	Eucalyptus grandis	EG0010G18440	142	218	548	895		
	Eucalyptus grandis	EG0011G25610	157	218	548	865		
	Fragaria vesca	FV6G12020	148	218	545	865		
	Glycine max	GM01G25890	142	218	548	877		
	Glycine max	GM03G17001	142	218	514	782		
	Glycine max	GM07G16740	142	218	548	874		
	Glycine max	GM18G41270	142	218	548	874		
	Gossypium raimondii	GR06G16940	157	218	545	865		
	Malus domestica	MD03G010010	142	218	545	865		
	Malus domestica	MD12G016190	142	218	545	865		
	Manihot esculenta	ME06244G00010	136	218	539	772		
	Manihot esculenta	ME06244G00020	136	218	539	760		
	Manihot esculenta	ME06549G00080	136	218	545	865		
	Manihot esculenta	ME07268G00020	136	218	539	922		

	Manihot esculenta	ME07268G00030	136	218	545	865		
	Medicago truncatula	MT4G019580	142	218	548	874		
	Medicago truncatula	MT7G065080	127	218	548	859		
	Prunus persica	PPE_002G23260	136	218	548	826		
	Prunus persica	PPE_006G27140	139	218	545	868		
	Populus trichocarpa	PT06G09200	136	218	545	865		
	Populus trichocarpa	PT16G10350	136	218	545	865		
	Solanum lycopersicum	SL10G084940	148	218	545	844		
	Solanum lycopersicum	SL10G084950	139	218	545	874		
	Solanum tuberosum	ST10G023870	139	218	539	874		
	Theobroma cacao	TC0005G02830	145	218	545	862		
	Theobroma cacao	TC0010G03040	145	218	545	883		
	Thellungiella parvula	TP4G20210	145	218	545	847		
	Thellungiella parvula	TP5G08440	145	218	545	883		
	Arabidopsis lyrata	AL4G27340	106	218	557	868		
	Arabidopsis thaliana	AT2G40460	106	218	557	871		
	Amborella trichopoda	ATR_00256G00010	212	218	557	913		
	Brassica rapa	BR03G20350	106	218	557	874		
	Brassica rapa	BR04G24210	106	218	557	868		
	Citrullus lanatus	CL07G04840	103	218	569	871		
	Cucumis melo	CM00041G00980	198	569	871			
NPF5.1	*Carica papaya*	CP00019G00960	103	218	557	291	598	
	Capsella rubella	CRU_004G21980	106	218	557	868		
	Citrus sinensis	CS00414G00250	103	218	557	877		
	Eucalyptus grandis	EG0007G24920	103	218	557	877		
	Fragaria vesca	FV7G30680	115	218	557	886		
	Glycine max	GM02G42740	109	59	557	467	350	
	Glycine max	GM04G39870	103	218	557	874		
	Glycine max	GM06G15020	103	218	557	874		
	Glycine max	GM11G35890	106	218	557	883		

	Glycine max	GM18G02510	106	218	557	886		
	Gossypium raimondii	GR10G21720	103	218	557	874		
	Gossypium raimondii	GR11G21090	103	218	557	874		
	Lotus japonicus	LJ1G017130	103	218	557	841		
	Lotus japonicus	LJ1G017150	103	218	557	861		
	Lotus japonicus	LJ6G004350	106	218	557	880		
	Malus domestica	MD00G408070	109	218	557	892		
	Malus domestica	MD04G002630	109	218	557	892		
	Manihot esculenta	ME00926G00130	103	218	557	868		
	Manihot esculenta	ME10689G00150	109	218	557	889		
	Medicago truncatula	MT3G072300	106	218	557	889		
	Prunus persica	PPE_002G25020	106	218	557	889		
	Populus trichocarpa	PT19G05580	103	218	557	898		
	Ricinus communis	RC29729G00650	103	218	557	880		
	Solanum lycopersicum	SL06G050900	106	218	557	865		
	Solanum tuberosum	ST06G009780	102	5	223	557	865	
	Theobroma cacao	TC0010G17220	103	218	557	877		
	Thellungiella parvula	TP4G22800	106	218	557	868		
	Vitis vinifera	VV13G09610	103	218	557	880		
NPF6.3	*Arabidopsis lyrata*	AL6G13130	226	224	578	850		
	Arabidopsis thaliana	AT5G13400	226	218	581	850		
	Brassica rapa	BR02G06350	220	218	581	349	501	
	Beta vulgaris	BV5G17080	112	218	515	832		
	Beta vulgaris	BV6G10460	365	218	581	835		
	Citrullus lanatus	CL07G14010	244	218	581	871		
	Cucumis melo	CM00031G02960	244	218	581	871		
	Carica papaya	CP00038G00620	232	218	581	862		
	Citrus sinensis	CS00330G00070	226	218	581	880		
	Eucalyptus grandis	EG0002G22800	115	218	551	832		
	Eucalyptus grandis	EG0007G14880	280	218	581	871		

	Fragaria vesca	FV0G14220	241	218	581	859		
	Glycine max	GM18G53710	226	218	581	871		
	Gossypium raimondii	GR05G19730	226	218	581	871		
	Lotus japonicus	LJ1G041460	226	218	581	877		
	Malus domestica	MD04G005700	238	218	581	877		
	Malus domestica	MD13G010750	211	218	581	869	59	
	Manihot esculenta	ME02264G00020	229	218	581	889		
	Manihot esculenta	ME03651G00110	106	218	551	832		
	Medicago truncatula	MT4G015080	136	218	551	829		
	Medicago truncatula	MT7G006540	223	218	163	418	895	
	Medicago truncatula	MT7G067010	106	218	551	832		
	Prunus persica	PPE_001G22150	238	218	581	877		
	Solanum lycopersicum	SL00G006820	456	581	925			
	Solanum tuberosum	ST11G017600	456	581	925			
	Theobroma cacao	TC0002G18600	238	218	581	874		
	Thellungiella parvula	TP6G30620	232	218	581	349	501	
	Vitis vinifera	VV01G01330	238	218	581	397	387	
NPF5.14	*Citrullus lanatus*	CL04G07660	163	218	554	430	453	
	Cucumis melo	CM00002G01170	151	218	554	436	450	
	Carica papaya	CP00056G00450	154	218	545	874		
	Eucalyptus grandis	EG0010G18440	142	218	548	895		
	Eucalyptus grandis	EG0011G25610	157	218	548	865		
	Fragaria vesca	FV6G12020	148	218	545	865		
	Fragaria vesca	FV7G28400	157	254	548	865		
	Glycine max	GM01G25890	142	218	548	877		
	Glycine max	GM11G34600	139	218	536	850		
	Glycine max	GM11G34613	127	218	536	850		
	Glycine max	GM11G34620	145	218	545	847		
	Glycine max	GM18G03770	133	218	545	874		
	Glycine max	GM18G03780	145	218	545	874		

	Glycine max	GM18G03790	148	218	548	844		
	Glycine max	GM18G03800	241	218	545	877		
	Gossypium raimondii	GR06G16940	157	218	545	865		
	Malus domestica	MD01G012940	160	290	545	862		
	Malus domestica	MD03G010010	142	218	545	865		
	Malus domestica	MD12G016190	142	218	545	865		
	Manihot esculenta	ME06549G00080	136	218	545	865		
	Manihot esculenta	ME07268G00030	136	218	545	865		
	Medicago truncatula	MT3G069440	133	218	545	877		
	Medicago truncatula	MT3G069500	139	218	545	844		
	Medicago truncatula	MT4G019580	142	218	548	874		
	Medicago truncatula	MT7G065080	127	218	548	859		
	Prunus persica	PPE002G23260	136	218	548	826		
	Prunus persica	PPE006G27140	139	218	545	868		
	Populus trichocarpa	PT06G09200	136	218	545	865		
	Populus trichocarpa	PT08G06110	151	218	542	886		
	Populus trichocarpa	PT16G10350	136	218	545	865		
	Solanum lycopersicum	SL10G084950	139	218	545	874		
	Solanum tuberosum	ST10G023870	139	218	539	874		
	Theobroma cacao	TC0005G02830	145	218	545	862		
	Theobroma cacao	TC0010G03040	145	218	545	883		

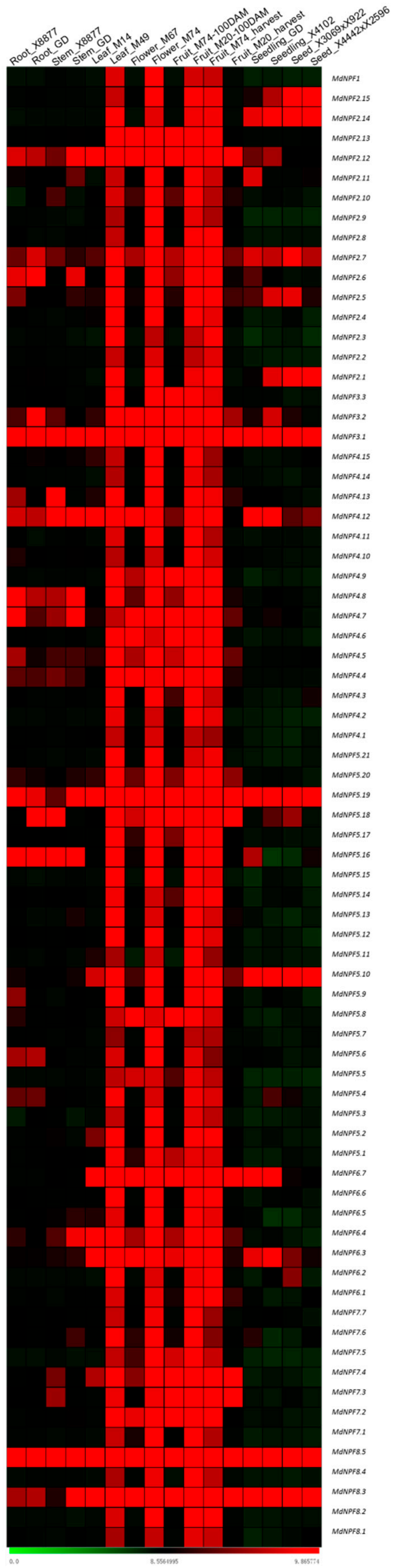

Supplementary Materials, Figure S2: Heat map of *MdNPF* expression in different tissues. Expression values were log2-transformed. Heat map was generated using TIGR MeV v4.8.1 software.

Supplementary Materials, Table S3 Primers used for quantitative real time RT-PCR amplifications.

Gene	Forward primer	Reverse primer
MdNPF2.5	TAGGGTTTGGCCTTTGTGTC	TGTCTGTCACTCGCCCATAG
MdNPF2.6	TGTTGGTTTCGGTCGTGTTA	TTCTGTGCTGCACGGTTAAG
MdNPF2.11	CCTTGACAAAGCTGCAATCA	TTTGTTGTTGCACCAGGGTA
MdNPF3.1	CAGTTCGACGAGAATGACGA	CAAGTGCCGGTAAAGATGGT
MdNPF4.4	TGCTGCCTACTTTGCCTTTT	CAGCCACAAAAACTTGAGCA
MdNPF5.1	AAGCCCTGCAAGAGATTTCA	ATGGTGCCCTTATTGAGACG
MdNPF5.13	CCAACGACCCACTTCTTCAT	TGACTTTGCTCCCTTGCTTT
MdNPF5.14	CTGGTTTGGGAAGGACTTGA	GAGAAGTGTCGTCAGCCACA
MdNPF5.16	GATCAGCAGGGAAATGGAAA	ATGCAAAGGCCATTGTTCTC
MdNPF5.19	ACATTTTGGGACTTGGCTTG	TTTTGGCTTTGCACTCCTCT
MdNPF6.3	TGCTCAAGTTTTGGTTGCTG	TCACAGTGCATAGCCTCCAG
MdNPF6.5	GGCCAACCAGGTTTCACTAA	ACCCACCAGGAAGAACTGTG
MdNPF6.7	GCTGGCTGAGGAGTAACCTG	ACTCCTTTTCGCCACTAGCA
MdNPF8.1	CTCATGGATGTTCGTGTTGG	TCCAAAACTGCAGCACAGAC

Supplementary Materials, Table S4: Primers used for gene-cloning.

Gene	Forward primer	Reverse primer
MdNPF2.5	ATGGATGGCTCCCTCTCCCACGAT	CTAGCTATCTGGGTCAGGACTAC
MdNPF2.6	ATGGATGAGTCCCTCTCCCACGCC	CTAGCTATCTGGATCAGGACTAC
MdNPF2.11	ATGCTGATACTAACCCTAACAGCG	TCAAACAAGATGTTTCTCAGATT
MdNPF3.1	ATGGAACATATTGAGGAGAAAGGC	TTTTCCGGATCCAGCACCTTCTT
MdNPF4.4	ATGGAACAAAGGAGGCAGAACCAC	TTATGGTATATTATCATCCAGTC
MdNPF5.1	ATGGAAGCCAAAGCTGCTGGTTAC	TTAGACTTGTAGTCCAAGTGGAG
MdNPF5.13	ATGGAAGAAAGCAAGGGAGCAAAG	TCATGGGATCGGGCCTCCATTTT
MdNPF5.14	ATGGAGCAAGAAATGGAAAAGAGA	CTAAGCCATAGATTGATCAATTC
MdNPF5.16	ATGGAGAGATTTGCGTACAAAGGA	TTATTTACAGGTTTCATTTTCCA
MdNPF5.19	ATGGCCGCTTCTCCCTGCCAATCC	TTACAAGGCGTGGGATTTATAAA
MdNPF6.3	ATGACACCAGCTAGTTTAGATGGA	CTAACCACTGAGGGGTTGTTCTT
MdNPF6.5	ATGGAGGGAGGGAAGATGGGTTGG	TTATGCACCATTAGCAGAAGCCG
MdNPF6.7	ATGGTTTTGATCTCAAGGAAAGGT	CTAAGGCCCTTCCTTTGCCTCAA
MdNPF8.1	ATGGCAGAAGATGATATGCTCGCC	TCAACGCAGAGTCCCGACGGTCT
